# HIV-1 capsid is involved in post-nuclear entry steps

**DOI:** 10.1186/s12977-016-0262-0

**Published:** 2016-04-23

**Authors:** Nan-Yu Chen, Lihong Zhou, Paul J. Gane, Silvana Opp, Neil J. Ball, Giuseppe Nicastro, Madeleine Zufferey, Cindy Buffone, Jeremy Luban, David Selwood, Felipe Diaz-Griffero, Ian Taylor, Ariberto Fassati

**Affiliations:** Division of Infection and Immunity, University College London, Cruciform Building, 90 Gower Street, London, WC1E 6BT UK; Division of Infectious Diseases, Department of Internal Medicine, Chang Gung Memorial Hospital, Chang Gung University College of Medicine, 5 Fuhsing Street, Kueishan, Taoyuan, 333 Taiwan; Medicinal Chemistry Group, University College London, Cruciform Building, Gower Street, London, WC1E 6BT UK; Department of Microbiology and Immunology, Albert Einstein College of Medicine, Bronx, NY 10461 USA; Mill Hill Laboratory, The Francis Crick Institute, The Ridgeway, Mill Hill, London, NW7 1AA UK; Department of Microbiology and Molecular Medicine, University of Geneva, 1 Rue Michel Servet, CH-1211 Geneva, Switzerland; Program in Molecular Medicine, University of Massachusetts Medical School, 373 Plantation Street, Biotech 2, Suite 319, Worcester, MA 01605 USA; Department of Biochemistry and Molecular Pharmacology, University of Massachusetts Medical School, 373 Plantation Street, Biotech 2, Suite 319, Worcester, MA 01605 USA; Genome Damage and Stability Centre, University of Sussex, Science Park Road, Falmer, Brighton, BN1 9RQ UK; Chemical Computing Group, St. John’s Innovation Centre, Cowley Road, Cambridge, CB4 0WS UK

**Keywords:** HIV-1, Capsid, Nucleus, Integration, Coumermycin-A1, Nup153, Nucleoporins, Uncoating

## Abstract

**Background:**

HIV-1 capsid influences viral uncoating and nuclear import. Some capsid is detected in the nucleus but it is unclear if it has any function. We reported that the antibiotic Coumermycin-A1 (C-A1) inhibits HIV-1 integration and that a capsid mutation confers resistance to C-A1, suggesting that capsid might affect post-nuclear entry steps.

**Results:**

Here we report that C-A1 inhibits HIV-1 integration in a capsid-dependent way. Using molecular docking, we identify an extended binding pocket delimited by two adjacent capsid monomers where C-A1 is predicted to bind. Isothermal titration calorimetry confirmed that C-A1 binds to hexameric capsid. Cyclosporine washout assays in Jurkat CD4+ T cells expressing engineered human TRIMCyp showed that C-A1 causes faster and greater escape from TRIMCyp restriction. Sub-cellular fractionation showed that small amounts of capsid accumulated in the nuclei of infected cells and C-A1 reduced the nuclear capsid. A105S and N74D capsid mutant viruses did not accumulate capsid in the nucleus, irrespective of C-A1 treatment. Depletion of Nup153, a nucleoporin located at the nuclear side of the nuclear pore that binds to HIV-1 capsid, made the virus less susceptible to TRIMCyp restriction, suggesting that Nup153 may help maintain some integrity of the viral core in the nucleus. Furthermore C-A1 increased binding of CPSF6, a nuclear protein, to capsid.

**Conclusions:**

Our results indicate that capsid is involved in post-nuclear entry steps preceding integration.

**Electronic supplementary material:**

The online version of this article (doi:10.1186/s12977-016-0262-0) contains supplementary material, which is available to authorized users.

## Background

Early post-entry events in the HIV-1 life cycle are still poorly understood, yet they may reveal important host–pathogen interactions and provide novel therapeutic targets. Following HIV-1 entry into cells, capsid proteins are shed from the conical core, a step called uncoating, and the viral RNA genome is reverse transcribed into a double stranded DNA molecule. Although different models have been proposed [1, 2], a consensus is emerging based on biochemical, microscopy and genetic approaches suggesting a stepwise uncoating of the viral core along with reverse transcription and intracellular trafficking [3–10]. Interestingly, uncoating has been shown to influence the early steps of HIV-1 infection. For example, certain mutations in the capsid protein (E45A, R132A, Q219A), which perturb core stability, impair reverse transcription [3]. Capsid Mutations Q63/Q67A and T54A/N57A show delayed uncoating and are defective in nuclear import and integration [11–13]. Some capsid mutations promote autointegration of the viral genome [14] whereas other changes in capsid or Gag alter the distribution of HIV-1 integrations sites [15, 16].

It is not always clear how capsid impacts on such steps. In some cases, capsid mutations prevent binding to specific host factors such as CPSF6 and nucleoporin Nup153 [17–23]. In other cases, the intrinsic stability of the viral core may be changed. Yet we detected low amounts of HIV-1 capsid inside nuclei of infected CD4 T cells [24], an observation later confirmed and extended by several groups [25–27], suggesting a more direct role for capsid in post-nuclear entry steps. Supporting this notion, capsid was shown to bind to nucleoporins Nup153 [18, 19, 22, 28] and Nup358, via the CypA binding domain [15, 29, 30], although the relevance of this interaction remains unclear [31]. Depletion of Transportin 3 (TNPO3), a nuclear transport receptor, impinges on both pre- and post-nuclear entry steps of HIV-1 and its effects map to capsid and host factor CPSF6 [21, 23, 24, 32–35]. It has been proposed that TNPO3 stimulates a nuclear uncoating step that promotes integration [24]. Recently, capsid was shown to be the target of a novel post-nuclear entry restriction to SIV infection [36] and to permit greater access of the HIV-1 PIC deeper into the nuclei [27]. Furthermore, by binding to CPSF6, capsid directs HIV-1 integration into transcriptionally active chromatin regions [37]. However the sequence of post-nuclear entry events dependent on capsid remain largely unknown.

Several small molecules that inhibit HIV-1 infection by targeting capsid have been described [38–45]. At least three of these molecules impair the early stages of the viral life cycle: PF-74, BI-1 and C-A1. PF-74 inhibits reverse transcription at high doses (>10 μM), possibly by inducing destabilization of the capsid core and premature uncoating [18, 23, 39, 46]. At lower doses (<8 μM), PF-74 is a competitive inhibitor of CPSF6 and Nup153 interaction with capsid [18, 28] and appears to act post-nuclear entry [26]. BI-1, which binds to capsid in a different mode, appears to stabilize the HIV-1 core, inhibiting nuclear transport of HIV-1 PICs without altering reverse transcription [18, 40] although there is also evidence that BI-1 might destabilize the core by competing with CPSF6 [47]. C-A1 is an antibiotic targeting gyrase B [48], which also impairs HIV-1 integration by an undefined mechanism [38]. Here we show that C-A1 impairs HIV-1 integration in a capsid-dependent way without affecting reverse transcription. C-A1 lowers the amount of capsid detected in the nuclei and at the same time promotes binding of CPSF6, a nuclear protein, to capsid. We also found that, similar to C-A1, depletion of Nup153 promotes greater rescue from restriction mediated by TRIMCyp. Our data suggest that capsid is functionally important for events preceding integration and that completion of HIV-1 uncoating may take place inside the nucleus.

## Results

### C-A1 inhibits HIV-1 integration

**Fig. 1:**
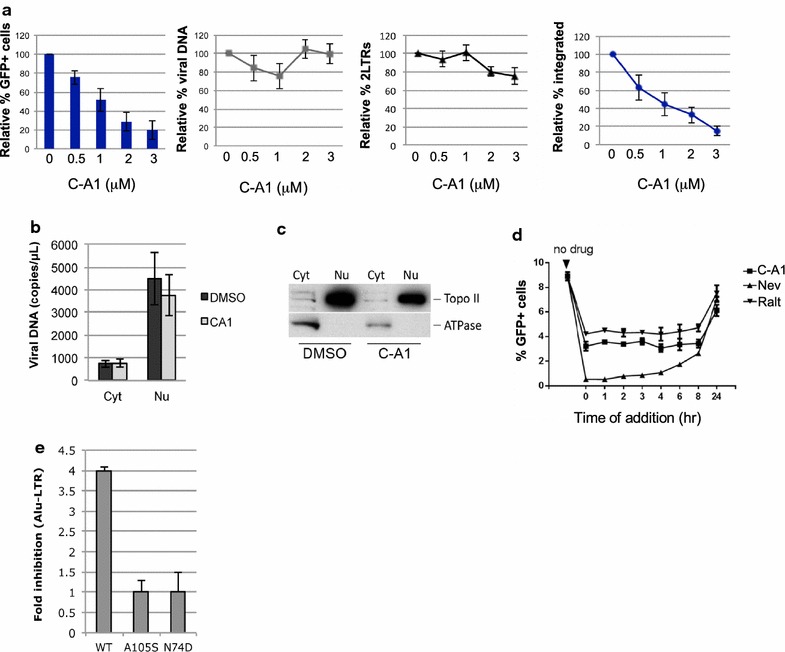
C-A1 inhibits HIV-1 integration and capsid mutations confer resistance to the drug. **a** Jurkat cells were infected with HIV-1_GFP_ at an MOI of 0.05–0.1 in the presence of the indicated concentrations of C-A1 and analyzed by FACS 24 h later to determine the percentage of infected cells, the amount of viral DNA and 2LTR circular DNA. The amount of integrated viral DNA was determined by Alu-LTR TaqMan qPCR 10 days post-infection. **b** Jurkat cells were infected with HIV-1_GFP_ at the same MOI in the presence or absence of 3 μM C-A1 and fractionated into a cytoplasmic and a nuclear fraction 16 h post-infection. The amount of viral DNA was measured by TaqMan qPCR in each fraction. **c** The quality of the fractionation was assessed by Western blot to detect DNA Topoisomerase II (Topo II) and Na/K ATPase (ATPase); representative of three experiments. **d** Time of addition assay. Jurkat cells were infected with HIV-1_GFP_ at an MOI of 0.1 and the compounds were added at the indicated time points after infection (time 0). Cells were analyzed by FACS 36 h post-infection. **e** Jurkat cells were infected with HIV-1_GFP_ WT, A105S or N74D capsid mutants at an MOI of 0.1 in the presence of 3 μM C-A1 and the quantity of integrated viral DNA was measured by Alu-LTR TaqMan qPCR 1 week post-infection. Average values ±SD of three independent experiments are shown in (**a**, **b**, **e**)

We previously reported that C-A1 inhibited HIV-1 integration [38]. To confirm this finding, we infected Jurkat CD4+ T cells with a single cycle VSV-G pseudotyped HIV-1 vector expressing GFP (HIV-1_GFP_) in the presence of increasing concentrations of C-A1. Cells were infected at an MOI of 0.05–0.1 then analyzed by FACS and Taqman qPCR at 24 h and 10 days post-infection (for integrated provirus). C-A1 significantly inhibited HIV-1 infection in a dose dependent way with an IC_50_ of 1 μM, however it did not affect reverse transcription or synthesis of 2LTRs circular DNA forms, a hallmark of nuclear entry [49] (Fig. 1a). In contrast, C-A1 reduced HIV-1 integration as measured by Taqman Alu-qPCR (Fig. 1a). We also fractionated cells infected with HIV-1_GFP_ in the presence or absence of 3 μM C-A1 and measured the amount of viral DNA in the cytoplasmic and nuclear fractions. In agreement with previous results [50], viral DNA was mostly nuclear 16 h post-infection and C-A1 did not change its distribution (Fig. 1b). DNA Topoisomerase II, a nuclear protein, and Na/K ATPase, a cytoplasmic and membrane protein, were used as markers of successful fractionation [24] (Fig. 1c).

We performed time of addition assays to evaluate by a different method the step of the HIV-1 life cycle blocked by C-A1. The rationale behind this assay is that the compound cannot block a step of the viral life cycle if added after the step has been completed. Inhibitors of reverse transcription and integration were used in parallel to define the temporal dynamics of each step [51]. Drugs were added at the indicated time point after infection, removed after 30 h and cells analysed by FACS 24 h later. This assay did not detect the effect of C-A1 on viral gene expression, which is reversible [38]. The reverse transcription inhibitor nevirapine started losing activity when added 4–6 h post-infection. The integration inhibitor Raltegravir [52] and C-A1 both started losing activity when added 8 h post-infection (Fig. 1d), confirming that C-A1 inhibits HIV-1 at the integration step.

To test if the effect of C-A1 was capsid-specific, we used a virus bearing the A105S capsid mutation. This capsid mutation was selected after passaging HIV-1 in the presence of C-A1 and conferred resistance to the drug when introduced into HIV-1 NL4.3 [38]. In parallel, we also tested the N74D mutation, which confers partial resistance to the small compound PF-74 [39]. A105S and N74D capsid mutants were both resistant to the integration block mediated by C-A1 (Fig. 1e). Taken together, these results demonstrated that C-A1 blocks HIV-1 integration in a capsid-specific way.

### C-A1 interacts with HIV-1 capsid

**Fig. 2:**
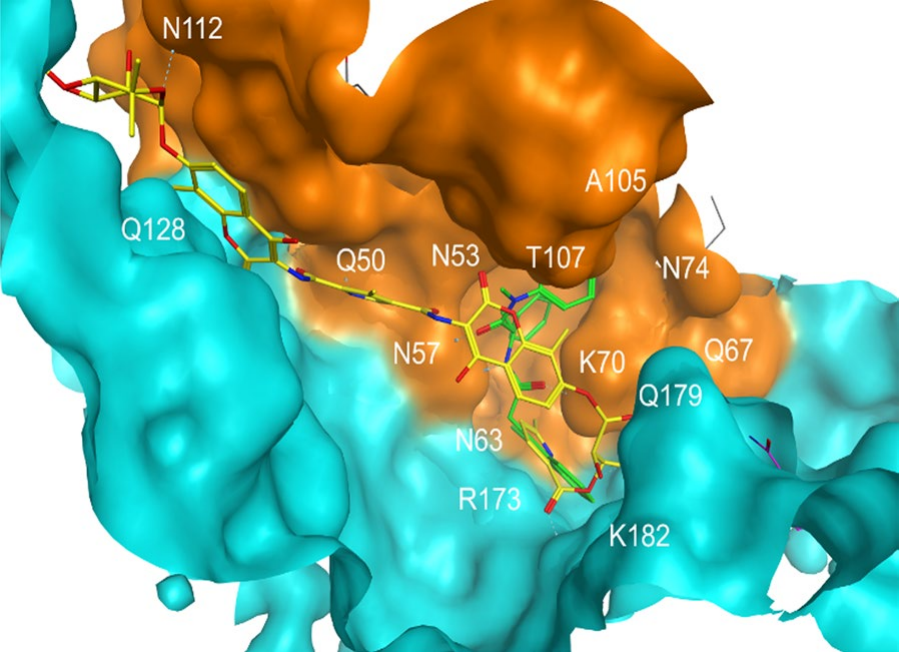
Molecular docking of C-A1 (*in yellow*) in HIV-1 hexameric capsid structure 4XFZ.pdb with bound PF-74 (*in green*) using the Compute: SiteFinder option in MOE software. A single C-A1 conformation is shown bound to capsid. The two subunits of capsid are depicted in *brown* and *turquoise* respectively. The position of key residues is indicated

The results shown in Fig. 1 strongly suggested that capsid is the primary target of C-A1. To examine this notion further, we performed molecular docking using the newly described native and hydrated hexameric HIV-1 capsid structure bound to PF-74 (4XFZ.pdb) [53]. This structure is well resolved in the region of helices 8 and 9 (H8/H9), where PF-74 binds, and it is in the open conformation, making it much better suited for docking of C-A1. We identified an extended binding pocket bounded by two adjacent monomers from the same hexameric ring structure using the Compute: SiteFinder option in MOE [54]. C-A1 docked at the site although most poses had one pyrrole exposed to solvent with minimal contacts with the protein. The C-A1-binding groove identified was similar but more extended than the binding site for PF-74 [39], BI-1 [40] and a CPSF6 peptide [18, 28, 40]. The groove is formed by both capsid subunits and includes Asn53, Asn57, Gln63, Lys70, Thr107 and Gln112 of subunit A, and Glu128, Arg173, Gln179, Lys182 of subunit B (Fig. 2; Additional file 1: Fig. S1). PF-74 occupies a smaller region of the cleft delimited by Asn57, Gln63, Lys70 and Thr107 of the A subunit and Arg173, Gln179 and Lys182 of the B subunit (Fig. 2; Additional file 1: Fig. S1).

The A105S and N74D mutations confer resistance to C-A1, suggesting that these residues are important for the antiretroviral effect of the drug. Ala105 is partially buried by Thr107 so direct contact with CA-1 is not observed (Fig. 2; Additional file 1: Fig. S1), but as this is a pinch-point in the binding groove, any mutation to a larger amino acid will restrict space in this region, presumably reducing the binding affinity of C-A1 (Fig. 2). Alternatively, such mutations may exert an indirect effect by changing the way C-A1 binds to capsid or abolishing binding to host-cofactors [20].

**Fig. 3:**
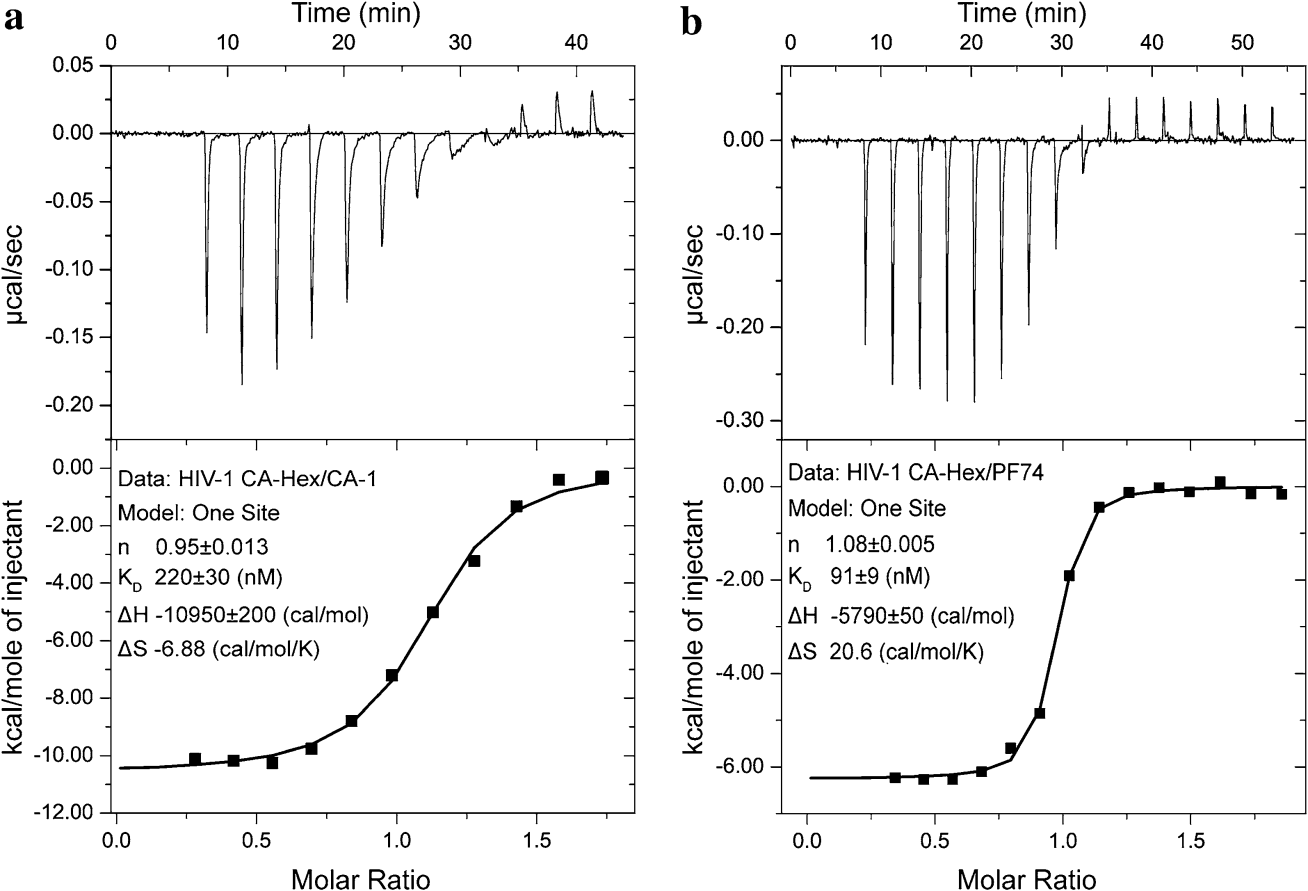
Analysis of drug–CA interactions. The interaction of C-A1 (**a**) and PF-74 (**b**) with HIV-1 CA hexamers was quantified by ITC. The *top panels* show raw thermograms and the *bottom panels* show the titration data along with best line of best fit and the fitted parameters (*inset*)

To test more directly the molecular docking predictions, we performed isothermal titration calorimetry (ITC) using disulphide linked HIV-1 CA hexamers. These data (Fig. 3) reveal that C-A1 binds to hexameric CA with a stoichiometry of 1 CA-1 per CA monomer and with a dissociation constant (Kd) of 220 nM, comparable with 90 nM observed for the PF74–hexamer interaction.

### C-A1 decreases sensitivity to TRIMCyp in CD4+ T cells

The C-A1 binding pocket in capsid is important for correct CA–CA intermolecular interactions and formation of the hexameric lattice that defines the mature HIV-1 core [55]. We hypothesized that C-A1 might perturb uncoating of the viral core, either by inducing its premature disassembly or by stabilizing it. To test this hypothesis, we performed cyclosporine (CsA) washout assays [4, 5, 56, 57]. This assay exploits the restriction factor TRIMCyp, which targets capsid directly at the cyclophilin A (CypA) loop, blocking infection [58]. In TRIMCyp expressing cells, HIV-1 is restricted but addition of CsA prevents TRIMCyp binding to capsid. CsA is added at the time of infection to prevent TRIMCyp restriction and then is removed at different time points. The effect of CsA is reversible so when the drug is removed, TRIMCyp is again able to restrict infection, as long as enough capsid proteins remain associated with the reverse transcription complex (RTC). Because CsA is removed at different time points, it is then possible to estimate when, in the course of infection, enough capsid has been shed such that TRIMCyp can no longer restrict the virus (Additional file 2: Fig. S2) [59]. In other words, this assay indirectly measures loss of capsid by determining for how long the virus remains susceptible to TRIMCyp restriction. We, and others, interpret the loss of TRIMCyp restriction over time as “uncoating” [5, 57]. Though indirect, the CsA washout assay has the advantage of detecting infectious or productive events rather than the bulk of the viral population and has been successfully used to measure the kinetics of HIV-1 uncoating in OMK cells, which naturally express TRIMCyp, and HeLa cells expressing OMK TRIMCyp [5, 57]. However the kinetics of HIV-1 uncoating appear to be cell type dependent [4], hence we have adapted the assay in CD4+ Jurkat T cells stably expressing an engineered form of the human TRIM5α protein fused with CypA at position S322 (T5Cyp) or a mutant version that does not bind to capsid and cannot restrict infection (H126Q) [60].

To establish the CsA washout assays in these cells, titrations were conducted first to determine the linearity of the signal. Cells were infected with increasing amounts of HIV-1_GFP_ and analysed by FACS 48 h later. T5Cyp cells showed a 10-fold restriction compared to H126Q cells with an almost linear dose–response curve within the 0.3–3 % infection range (T5Cyp cells) or 0.5–19 % range (H126Q) (Additional file 3: Fig. S3). The same results were obtained with N74D and A105S HIV-1_GFP_ (Additional file 3: Fig. S3). Therefore these Jurkat cells were infected at a low MOI (0.01–0.03) to prevent saturation of T5Cyp and maintain linearity of the readout.

Next, we tested the ability of CsA to rescue infection. To this end, T5Cyp and H126Q cells were infected in parallel with a fixed amount of HIV-1_GFP_ in the presence of increasing concentrations of CsA. In H126Q cells, CsA weakly inhibited infection whereas in T5Cyp cells it rescued infection at concentrations >0.6 μM reaching a plateau at about 1.25 μM (Additional file 3: Fig. S3). By gating on the live cell population, it was found that CsA started to be toxic at concentrations >1 μM (Additional file 3: Fig. S3). Therefore, it was decided to use 1 μM CsA in the assay, which afforded good rescue of infection without toxicity.

Preliminary CsA washout assays were carried out as described in Additional file 2: Fig. S2. TRIMCyp and H126Q cells were infected on ice with HIV-1_GFP_ virus (at an MOI 0.01–0.03) in the presence of 1 μM CsA or 3 μM C-A1. Cells were then spinoculated at 4 °C to synchronize infection and CsA was removed by media change at different time points (0, 1, 2, 3, 4, 6 and 8 h after spinoculation). The percentage of infected GFP+ cells was measured by FACS 48 h later. Approximately 70 % cells were alive as determined by gating on the untreated population.

Neither CsA nor C-A1 significantly affected the levels of infection over time in control H126Q cells (Additional file 4: Fig. S4), which was expected because C-A1 was washed out early hence could not inhibit infection on its own. In contrast, CsA significantly rescued infection in T5Cyp cells and the rescue effect was greater at later washout time points (Additional file 4: Fig. S4). This was consistent with the notion that CsA protected more viruses from TRIMCyp restriction if removed at a later time point. C-A1 on its own did not rescue infection. An extended assay showed that the rescue of infection mediated by CsA reaches a plateau at about 10 h (Additional file 3: Fig. S4), suggesting that, by that time, most virions probably were no longer sensitive to TRIMCyp, making CsA irrelevant.

**Fig. 4:**
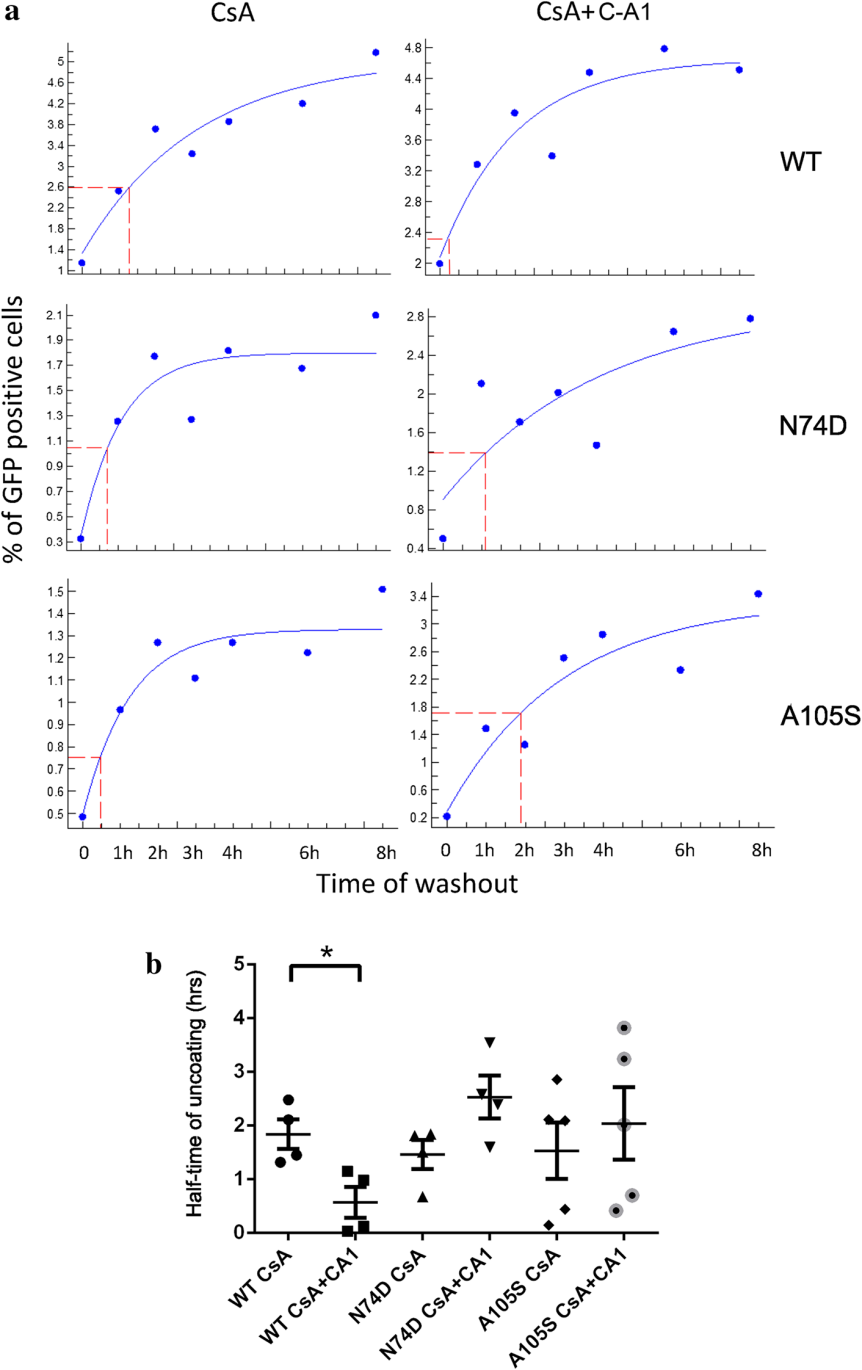
C-A1 reduces sensitivity to T5Cyp in CD4+ T cells. **a** CsA washout assays were performed on Jurkat cells stably expressing T5Cyp. Cells were infected with HIV-1_GFP_ WT, N74D or A105S mutants in the presence of 1 μM CsA alone or in combination with 3 μM C-A1. The drugs were washed out at the indicated time points and cells analyzed by FACS 48 h post-infection to calculate the percentage of infected (GFP+) cells. Some background infection was detected after spinoculation hence values on the Y-axis do not start at 0. Data were plotted using XLfit to determine the half time of uncoating (ToU_50_). The representative ToU_50_ of six experiments is shown (*red dashed line*). **b** *Plot* showing average values of ToU_50_ ± SEM of four independent experiments. *Each dot* on the graph represents an independent experiment (note that five experiments were carried out with the A105S mutant virus). *p < 0.02, statistical significance was calculated using Student’s unpaired, two-tailed *t* test

CsA washout assays were then performed with wild type (WT) or capsid mutant N74D and A105S HIV_GFP_ vectors to control for specificity. Infection levels, expressed as percentage of GFP+ cells, were measured for each time point by FACS and plotted using the software XLfit (ID Business Solutions Ltd.) to calculate the point at which 50 % infection levels were reached, relative to the 8 h time point (Fig. 4a). This is the point where 50 % RTC lose susceptibility to T5Cyp because productive infection takes place even if CsA is washed out. This point is hereafter called “the half-time of uncoating” or ToU_50_. The expectation is that if CA is shed faster from the RTCs, the ToU_50_ will be smaller whereas a greater ToU_50_ indicates slower uncoating. Data from four independent experiments were collected: the average ±SEM ToU_50_ for WT HIV-1_GFP_ was 110 ± 16 min, for the N74D virus was 88 ± 16 min and for the A105S virus was 92 ± 31 min (Fig. 4b). This is longer than ToU_50_ observed in OMK cells (40–70 min) [5], which may be due to the cell type used in our assays. Upon addition of C-A1, the ToU_50_ of WT HIV-1_GFP_ was significantly shortened to approximately 34 ± 17 min, whereas ToU_50_ appeared higher for N74D (151 ± 23 min) and A105S (122 ± 40 min) viruses, although the difference did not reach statistical significance (Fig. 4b). These data indicated that C-A1 makes the virus less susceptible to TRIMCyp, possibly by accelerating the loss of capsid in infected cells. This effect was dependent on specific targeting of CA.

### C-A1 reduces accumulation of capsid into the nucleus

**Fig. 5:**
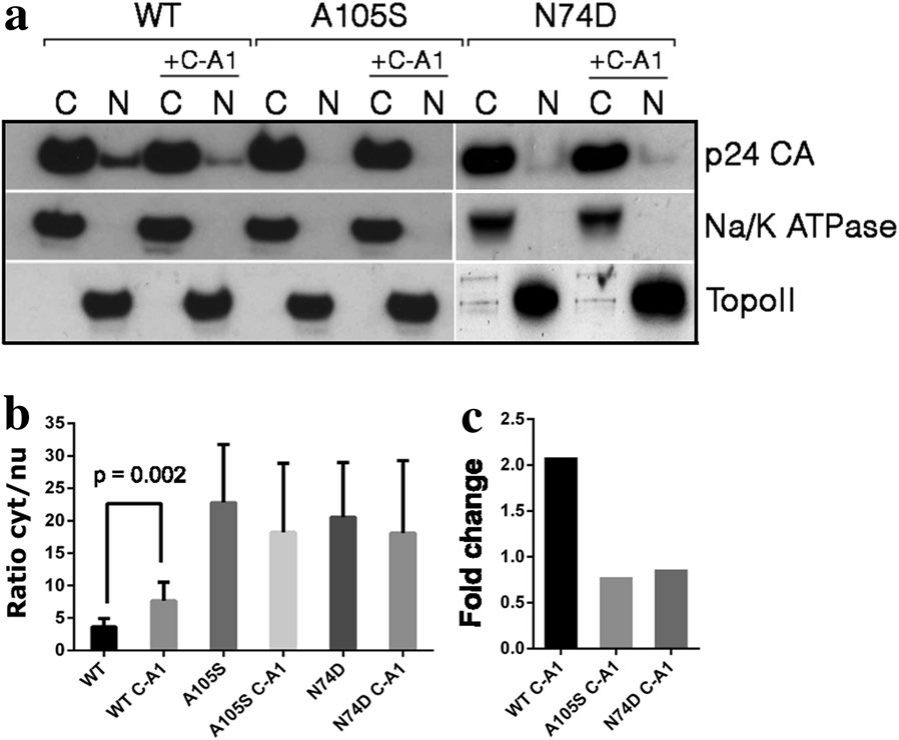
C-A1 reduces accumulation of capsid into the nucleus of infected cells. **a** Jurkat cells were infected at an MOI of 0.5 with HIV-1_GFP_ WT, A105S or N74D mutants in the presence or absence of 3 μM C-A1. Cells were fractionated 16 h post-infection and the distribution of capsid, Na/K ATPase and DNA Topoisomerase II (Topo II) in the nucleus and cytoplasm examined by Western blot. **b** The ratio of cytoplasmic versus nuclear capsid was calculated using ImageJ and normalized for Na/K ATPase (cytoplasmic fraction) or TopoII (nuclear fraction). Average values ±SD of at least four independent experiments are shown for WT virus and three experiments for N74D or A105S viruses. **c** Fold change in the cytoplasmic/nuclear ratio of capsid based on ImageJ quantification. Statistical significance was calculated using Student’s unpaired, two-tailed t-test

We returned to the issue of nuclear capsid and integration. We reasoned that C-A1, by stimulating uncoating, should reduce the amount of nuclear capsid associated with the PIC, which may affect integration. Thus we examined if C-A1 affected the distribution of capsid in the nucleus and cytoplasm of acutely infected Jurkat cells. Cells were infected with HIV-1_GFP_ at an MOI of 0.5 in the presence or absence of C-A1 and 16 h later fractionated into nuclear and cytoplasmic extracts, which were analyzed for the presence of capsid by Western blot (Fig. 5a). C-A1 had no effect on the overall amount of HIV-1 capsid detected in the cytoplasm, however it reduced nuclear accumulation of capsid and this effect was reproducible (Fig. 5b–c). The A105S and the N74D mutant viruses did not show significant accumulation of capsid into the nucleus, with or without C-A1, indicating that such viruses shed almost all of their capsid before nuclear entry (Fig. 5).

### Depletion of Nup153 affects sensitivity to T5Cyp

We sought further evidence that capsid in the nucleus was associated with a functional PIC and opted to exploit Nup153 for this purpose.

Nup153 has three domains, an-N-terminal domain that anchors it to the nuclear ring region of the NPC, a central domain that contains four zinc-fingers regions and C-terminal domain that is rich in FG repeats [61–63]. Nup153 is located on the nuclear side of the nuclear envelope [61–65] and its N-terminal domain interacts with Nup50 [66], located at the nuclear basket, and Tpr, forming the nuclear filaments [67]. The C-terminal domain of Nup153 is flexible and it is sometimes observed stretching towards the cytoplasmic face of the NPC [68]. Nup153 binds to HIV-1 capsid and has been proposed to modulate uncoating and integration [19, 22]. Given its intranuclear localisation and its ability to bind to hexameric HIV-1 CA, we decided that Nup153 would be a good tool to investigate if functional nuclear PICs contain CA. To this end, we decided to perform CsA washout assays in cells depleted of Nup153. If Nup153 promotes virus uncoating at the NPC, then its depletion should result in more capsid bound to the virus and greater susceptibility to TRIMCyp restriction. In contrast, if Nup153 helps maintaining capsid at the viral core, its depletion should result in more shedding of capsid and lower susceptibility to TRIMCyp restriction (greater rescue of infection).

**Fig. 6:**
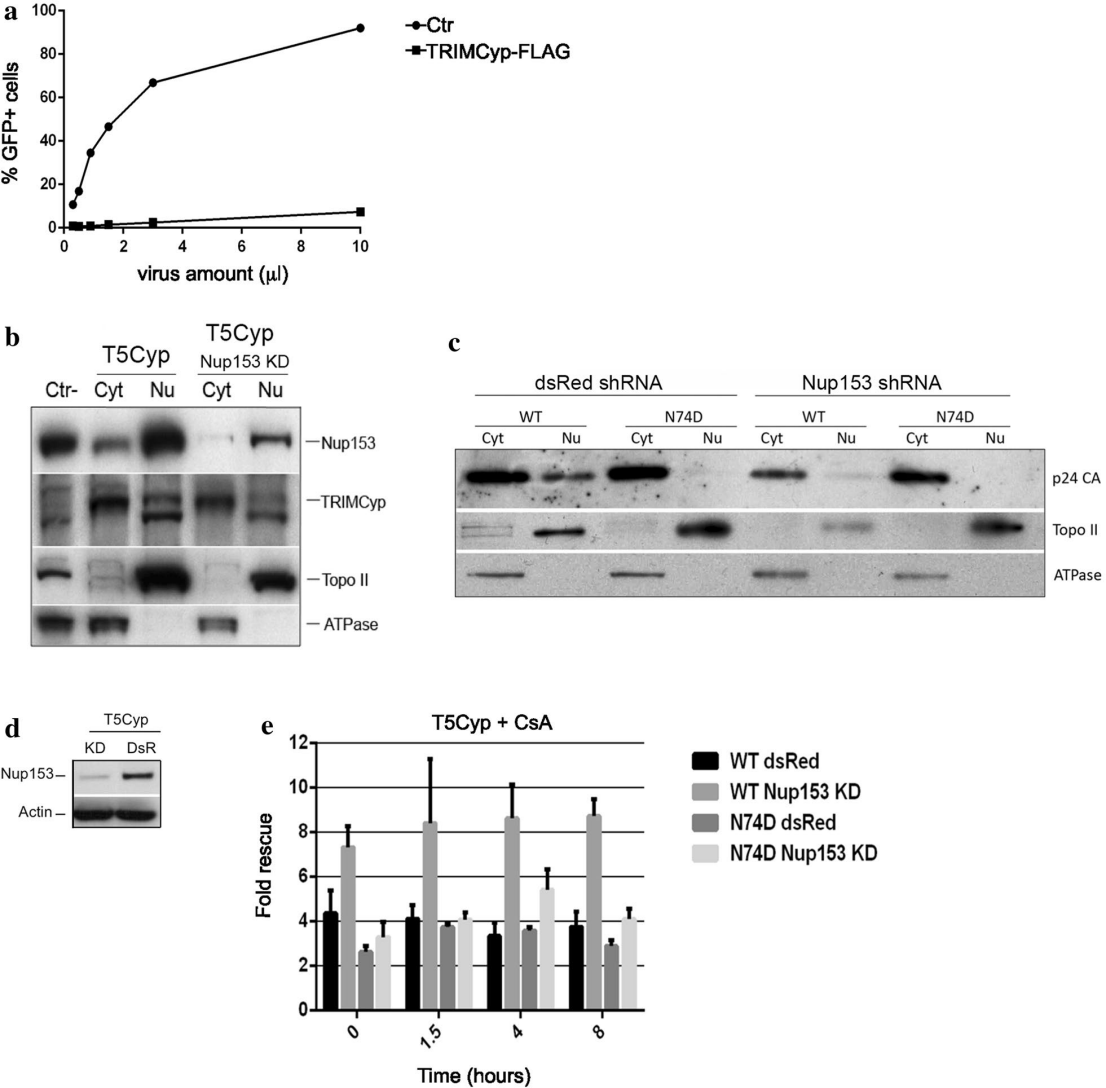
Depletion of Nup153 affects sensitivity to T5Cyp in CD4+ T cells. **a** Jurkat cells stably expressing a Flag-tagged TRIMCyp or an empty vector (Ctr) were infected with increasing doses of HIV-1_GFP_ and analyzed by FACS 48 h later. **b** Cells stably expressing TRIMCyp were depleted of Nup153 using shRNA. Cells were fractionated into nuclear and cytoplasmic fractions and analyzed by Western blot to detect the distribution of TRIMCyp, Nup153, DNA Topo II and Na/K ATPase. *Cyt* cytoplasmic fraction, *Nu* nuclear fraction, Ctr-, Jurkat cells not expressing TRIMCyp. **c** Cells depleted of Nup153 or control cells expressing an shRNA targeting the *Discosoma corallimorpharian* mRNA (dsRed) were infected with HIV-1_GFP_ at an MOI of 0.1 in the presence of CsA, fractionated into nuclear and cytoplasmic fractions and analyzed by Western blot to detect the distribution of capsid, DNA Topo II and Na/K ATPase. **d** Nup153 was depleted by shRNA in T5Cyp cells. An shRNA targeting DsRed (DsR) was used as control. **e** These cells were used to perform CsA washout assays upon infection with HIV-1_GFP_ WT or capsid mutant N74D. Cells were analyzed by FACS 48 h post-infection and the fold rescue of infection induced by CsA was plotted. Depletion of Nup153 enhanced rescue of infection significantly in T5Cyp cells and the effect was specific for HIV-1_GFP_ WT. Average values of rescue of infection ±SD are shown for three independent experiments

First, we tested if TRIMCyp can be detected in the nucleus, similar to TRIM5α [69]. We generated Jurkat cells stably expressing a C-terminus Flag-tagged TRIMCyp, which potently restricted HIV-1 infection (Fig. 6a). In these cells we also depleted Nup153 by shRNA and performed cell fractionation. Nup153 was mostly nuclear (Fig. 6b upper panel) although some Nup153 was detected in the cytoplasm, presumably due to small amounts of nuclear envelope contaminating the cytoplasmic fractions (Fig. 6b upper panel). TRIMCyp was mostly present in the cytoplasm, however TRIMCyp could also be detected in the nucleus in both control and Nup153 KD cells (Fig. 6b). The quality of the fractionation was confirmed by the distribution of Nup153, DNA Topo II and Na/K ATPase (Fig. 6b, lower two panels).

Second, we investigated the presence of nuclear capsid in T5Cyp cells depleted of Nup153. T5Cyp cells were infected in the presence of CsA, fractionated and examined by Western blot to detect capsid (Fig. 6c). In cells infected with WT virus, small amounts of capsid were detected in the nuclei of both control and Nup153 KD cells, albeit in Nup153 depleted cells the overall signal was weaker (Fig. 6c). In cells infected with the N74D virus, virtually no capsid was detected in the nuclei, irrespective of Nup153.

Having established that both restriction factor (TRIMCyp) and target (capsid) are detectable in the nuclei, we performed CsA washout assays (Fig. 6e, Additional file 5: Fig. S5). Depletion of Nup153 added an additional block to HIV-1 infection on top of T5Cyp-mediated restriction (Additional file 5: Fig. S5) but, notably, resulted in a greater rescue of infection relative to control DsRed KD cells [50] (Fig. 6e, Additional file 5: Figure S5). As such, the fold rescue of infection, measured as the ratio between infection levels in the presence or absence of CsA, was higher in Nup153 KD cells compared to DsRed KD T5Cyp cells (Fig. 6e). Furthermore, rescue of infection was clearly capsid-specific because it could not be detected with the N74D virus, which indeed showed no nuclear capsid upon cell fractionation (Fig. 6e, Additional file 5: Figure S5). This suggested that, in Nup153-depleted cells, more RTC/PICs escaped T5Cyp restriction thus Nup153 may help stabilize what is presumably a partially disassembled core navigating the NPC. These results also indicated that nuclear capsid is associated with functional PICs, which can be targeted and inactivated by T5Cyp.

### C-A1 promotes CPSF6 binding to HIV-1 capsid cores

**Fig. 7:**
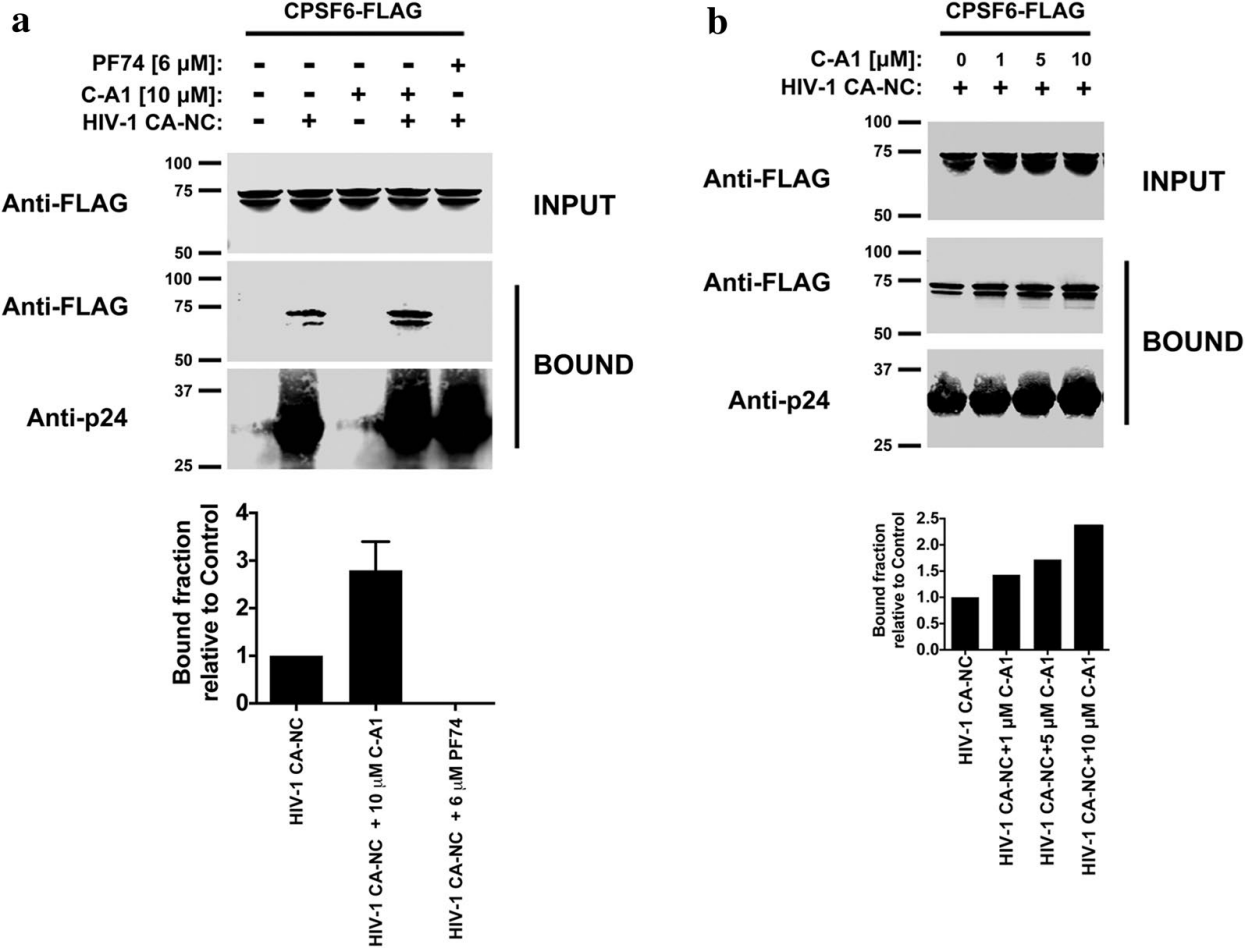
C-A1 enhances capsid binding to CPSF6. **a** Cellular extracts expressing CPSF6-FLAG were incubated with in vitro assembled HIV-1 CA–NC complexes at room temperature for 1 h in the presence of C-A1 or PF74. The mixtures were applied onto a 70 % sucrose cushion and centrifuged. INPUT represents the mixtures before being applied to the 70 % cushion. The INPUT was Western blotted using anti-FLAG antibodies. The pellet from the 70 % cushion (BOUND) was analyzed by Western blotting using anti-FLAG and anti-capsid antibodies. The results of three experiments were similar and standard deviations are shown. **b** Similar binding experiments were performed using increasing concentrations of C-A1. The results of three experiments were similar

The host factor CPSF6 is known to bind hexameric capsid both in vitro and in cells and influence HIV-1 infection [17, 18, 20, 21, 23, 28, 47, 70]. In physiological conditions, CPSF6 is a nuclear protein, which participates in cellular RNA processing (mainly poly site selection cleavage and polyadenylation) [71] and may guide the PIC towards active regions of chromatin [27]. Given that C-A1 binds to a cleft in capsid that in part overlaps with the CPSF6 binding site, we asked if C-A1 affected CPSF6 binding to capsid. We performed pull down assays by combining in vitro assembled CA–NC tubes and extracts from cells expressing Flag-tagged CPSF6. Remarkably, C-A1 increased CPSF6 binding to cores in a dose-dependent manner, whereas PF-74 showed the opposite effect (Fig. 7a, b), in agreement with previous reports [18, 28, 47]. These results suggested that C-A1 likely perturbs nuclear events regulated by both CPSF6 and capsid, resulting in an integration defect.

## Discussion

We previously suggested that C-A1 targeted capsid because serial passaging of HIV-1 in the presence of the drug resulted in the emergence of a drug-resistant virus with the A105S capsid mutation. This point mutation was necessary and sufficient to confer full resistance to the integration block mediated by C-A1 [38]. We have now provided strong evidence that C-A1 targets capsid. We identified another capsid point mutation, N74D, conferring resistance to C-A1. Molecular docking analysis identified a large cleft in the hexameric capsid structure where C-A1 was predicted to bind. ITC demonstrated that C-A1 binds to hexameric capsid with a Kd of 220 nM, which compares well with 90 nM observed for the PF74–hexamer interaction. Collectively, the data support the notion that C-A1 binds to hexameric capsid. Moreover, although, we have employed computational modelling and a co-crystal structure will ultimately be required to demonstrate the mode of C-A1 binding to capsid, our biochemical and biophysical data do support the notion for a CA-1 interaction that requires contributions from more than one monomer in the hexamer as is observed with CPSF6, Nup153 and PF-74 [18, 28].

It is intriguing how novel inhibitors of Hepatitis C virus replication have a symmetric chemical structure, similar to C-A1. Such inhibitors are also predicted to bind to a large cleft in the C-terminal domain of NS5A protein and perturb its dimerization [72]. Hence large and symmetric compounds such as C-A1 may provide a general platform to perturb dimerization or oligomerization of viral proteins.

C-A1 inhibits HIV-1 integration, which led us to explore the possible functional relationship between capsid and integration. We proceeded in a stepwise fashion by investigating the effect of C-A1 on capsid uncoating, nuclear accumulation and binding to CPSF6.

The activity of C-A1 was tested *in cellulo* by the CsA washout assay. To our knowledge, we were the first to perform CsA washout assays in CD4+ T cells stably expressing an engineered human TRIMCyp. We went to some length to establish the assay in CD4+ T cells because of their relevance for HIV-1 infection. The assay was successfully established, although working with suspension cells meant that analysis of very early time points (between 15 min and 1 h) was not possible. Repeated centrifugations to washout the drugs at very early time points prevented infection above background (not shown) and a minimum of 1 h incubation with the virus was necessary. Although we lost such early time points in our assay, HIV-1 uncoating in T cells has been reported to take hours rather than minutes [4]. In our assay we found that C-A1, in combination with CsA, reduces the sensitivity of HIV-1 to T5Cyp restriction. Because restriction depends on direct binding of T5Cyp to the CypA loop of HIV-1 capsid, the simplest interpretation of the results is that loss of restriction is caused by a loss of capsid, or uncoating. We exclude that C-A1 directly or indirectly inactivated T5Cyp because addition of the drug alone did not rescue HIV-1 infection in T5Cyp cells. We found that the ToU_50_ for HIV-1 in Jurkat cells was about 110 min, which is somewhat longer than the ToU_50_ reported in HeLa and OMK cells (40–70 min) [5, 57], however kinetics of uncoating are cell type dependent and slower in CD4+ T cells [4].

By performing cellular fractionation, we examined the downstream consequences of greater capsid disassembly induced by C-A1. We detected less capsid inside, or associated with, the nuclei of C-A1 treated cells compared to untreated cells. Two scenarios may explain this observation. Either free capsid proteins shed from the RTC/PIC reached the nuclei and were degraded faster than in the cytoplasm, or PICs in the nuclei contained less capsid following treatment with C-A1. We prefer the second possibility, which would be more consistent with previous genetic and imaging results implicating capsid in post-nuclear entry steps [24–27, 73]. However, the hypothesis that capsid remained associated with functional PICs in the nucleus required further validation.

To test this notion, we used the CsA washout assay in cells depleted of Nup153. The assay measures functional PICs, and the nuclear location of Nup153 means that any change in the susceptibility to T5Cyp restriction is likely to occur in the nucleus or at the nuclear side of the NPC. Post-nuclear entry restriction is not unprecedented. Fusion between the restriction factor Fv-1 and CypA resulted in chimeric proteins that maintained their specific ability to target capsid yet they restricted HIV-1 at post nuclear entry steps [74, 75]. Recently, a capsid-specific restriction of SIV infection was also shown to be post-nuclear entry [36].

We confirmed that depletion of Nup153 reduced HIV-1 infection in T5Cyp cells [30, 76]. Unexpectedly, we also found that depletion of Nup153 induced greater rescue of HIV-1 from T5Cyp restriction, suggesting that loss of Nup153 led to a greater loss of capsid. Thus Nup153 appears to stabilise capsid on functional PICs. The N74D mutant virus remained insensitive to Nup153 depletion, presumably because it had already lost almost all capsid by the time it entered the nucleus. Overall, the results supported the notion that capsid remains associated with functional PICs in the nuclear fraction. Some caution is required, however, because depletion of Nup153 might affect the nucleocytoplasmic distribution of host factors that stabilize, or destabilize, the viral core. Furthermore, depletion of Nup153 may result in the loss of Nup98 and Nup93 from the nuclear basket of the NPC [63], which may also indirectly affect HIV-1 capsid.

Regardless if Nup153 has a direct or indirect effect on core stability, we might need to reconsider some of the early steps of the HIV-1 life cycle: it was assumed that HIV-1 uncoating starts early post-infection and is completed well before the PIC enters the nucleus [77]. Although recent evidence confirmed that shedding of the capsid does indeed start early post-infection [27, 73, 78], uncoating may be completed after PIC nuclear entry [24]. This would fit with our recent data suggesting that the biophysical properties of the NPC barrier ensures that large cargos, such as the PIC, are efficiently transported across the pore provided that enough contact surface is available on the cargo to tip the fine-tuned Nup–Nup interactions and make the barrier permeable [79]. In this respect, capsid, which binds to Nups, may have an important, yet unappreciated, function.

How then can C-A1 affect integration? If capsid is required for efficient integration, the drug might act by reducing capsid associated with nuclear PICs. However this hypothesis is not fully convincing because viruses harboring the A105S and N74D mutations are insensitive to C-A1, they do not show accumulation of capsid in the nucleus yet they are capable of integrating. To clarify this issue, we tested the effect of C-A1 on the interaction between capsid and CPSF6. Remarkably, C-A1 stimulated binding of CPSF6 to capsid in a dose dependent way. It is presently unclear how C-A1 might enhance capsid binding to CPSF6 given that the C-A1 binding cleft is predicted to partly overlap with that one of PF-74 and BI-1. However C-A1 is a large and flexible molecule, which might fold to cross-link CPSF6 to capsid, given also that one of its pyrrolic rings may be exposed to solvent hence may be available for additional interactions.

We highlight the fact that CPSF6 is a nuclear protein in physiological conditions, and that tiny amounts of CPSF6 are detected in the cytoplasm only in cells lacking TNPO3 [21, 27]. Thus our results suggest that C-A1 may prevent capsid dissociation from CPSF6 in the nucleus. CPSF6 is known to stabilise the HIV-1 capsid core [21, 23] hence C-A1 may tether the PIC to CPSF6 negating further movement or maturation. This scenario would nicely explain why mutant viruses that shed their capsid before nuclear entry are insensitive to C-A1: they would not be tethered to CPSF6.

**Fig. 8:**
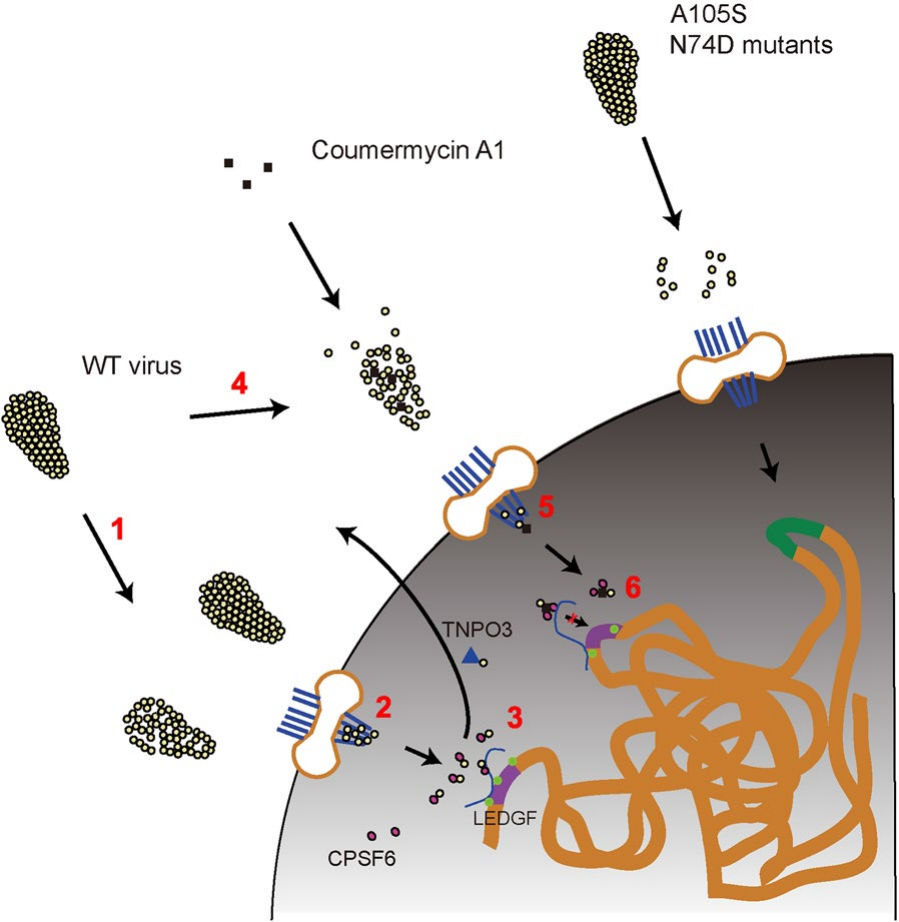
Model of C-A1 mode of action. (*1*) HIV-1 uncoating in the cytoplasm and at the nuclear pore. (*2*) A partially disassembled core is transported across the nuclear pore, reaching the nuclear basket where Nup153 helps maintaining the PIC structure. (*3*) The PIC is delivered onto chromatin domains rich in actively transcribing genes by binding to CPSF6. Some of these domains are in proximity of the nuclear pores [80, 81]. Uncoating is completed, possibly by TNPO3; capsid and CPSF6 are dissociated from the PIC, which binds to LEDGF to integrate in proximity of genes. (*4*) C-A1 perturbs this sequence of events by inducing greater uncoating and by affecting the PIC structure bound to Nup153 (*5*). When the PIC is delivered onto active chromatin domains, capsid cannot properly dissociate from CPSF6 such that the PIC remains tethered and is unable to proceed to integration (*6*). Mutant viruses N74D and A105S proceed through a capsid-independent pathway and are insensitive to C-A1

In conclusion, we propose a model that integrates the results presented here and recent data in the literature (Fig. 8). HIV-1 starts uncoating in the cytoplasm and at the nuclear pore. A partially disassembled core is transported across the nuclear pore, reaching the nuclear basket. Here, Nup153 helps maintain the PIC structure, which is translocated onto chromatin domains rich in active RNA processing by binding to CPSF6. These domains are often found in proximity of the nuclear pores [80, 81]. There, uncoating is completed, possibly by TNPO3 [24]; capsid and CPSF6 are dissociated from the PIC, which binds to LEDGF to integrate in proximity of actively transcribed genes. C-A1 perturbs this sequence of events by inducing greater uncoating and by affecting the PIC structure bound to Nup153. When the PIC is translocated onto active chromatin domains, capsid cannot properly dissociate from CPSF6 such that the PIC remains tethered and is unable to proceed to integration. This model is consistent with published imaging data showing that nuclear PICs contain capsid, which associates with CPSF6 inside the nuclei [27] and with data showing that capsid is important to target HIV-1 integration into active chromatin regions by binding to CPSF6 [37]. Mutant viruses N74D and A105S proceed through a capsid-independent pathway, which makes them insensitive to C-A1. However such mutant viruses do not occur in natural HIV-1 infections [82], suggesting that the capsid-dependent way of integration may be important in vivo.

## Conclusions

We conclude that the antibiotic Coumermycin-A1, by binding to HIV-1 capsid, perturbs the sequence of nuclear events leading to correct viral integration. We provide evidence for a post-nuclear entry step in the HIV-1 life cycle that can be targeted by a small molecule and define a new mechanism of action for Coumermycin-A1.

## Methods

### Chemical reagents

C-A1, PF-74 and CsA were purchased from Sigma and dissolved in DMSO at a final concentration of 5, 10 and 42 mM respectively.

### Docking studies

For identifying the binding cleft and initial docking of CA-1 the following software programs were used: FRED_receptor version 2.2.5 (2009) and FRED (Fast Rigid Exhaustive Docking) version 2.2.5 (2009) [Open Eye Scientific Software Inc., Santa Fe, NM 87507]. Subsequent docking was carried using the docking module within MOE (version 2014.09) [Chemical Computing Group Inc., Montreal, Quebec, Canada] [83]. The crystal complex 4XFZ.pdb was imported into MOE as a biological unit (i.e.; Symmetry biologic) giving the hexamer. The docking parameters used were: Forcefield—Amber10:EHT, Reaction Field for solvation, initial scoring London deltaG, refinement with BBV/WSA deltaG. A limited minimization of C-A1 and contacting side chains allowed H-bond partners to be fulfilled.

### ITC

C-terminally His-tagged HIV-1 CA A14C/E45C/W184A/M185A quadruple mutant was expressed in *E. coli* and purified by nickel affinity and gel filtration chromatography. Cross-linked hexamers were produced as previously described [84] and then exhaustively dialysed against ITC buffer (20 mM sodium phosphate pH 7, 150 mM NaCl). Prior to ITC, PF-74 and C-A1 were dissolved at 10 mM in DMSO, diluted to their final concentrations (PF-74—30 μM, C-A1—12 μM) in ITC buffer. The concentration of C-A1 in the experiment was confirmed by 1D ^1^H NMR through comparison of methyl resonances with those of a Trimethylsilyl propanoic acid standard reference. The monomer concentration of CA-hexamers was 390 μM for PF-74 and 120 μM for C-A1 titrations respectively. DMSO was also added to the hexamers to match the final DMSO concentration of the drug. ITC experiments were carried out at 293 K by titrating HIV-1 CA hexamers into the cell containing either C-A1 or PF-74 at 180 s intervals (3 μL injections for C-A1, 1.5 μL injections for PF-74). Heats of dilution for the hexamers were measured and subtracted from the titrations prior to data fitting. Binding isotherms were fitted to a single-site model using ORIGIN (MicroCal), where the hexamer concentration was expressed in terms of the monomeric equivalent.

### Cells and viruses

293T cells were grown in Dulbecco’s modified Eagle’s medium (DMEM) (Gibco Labs, Paisley, UK) supplemented with 10 % foetal calf serum (FCS) (Helena Bioscience, Newcastle, UK) and 2 mM glutamine at 37 °C in 5 % CO_2_. Jurkat cells were grown in RPMI medium supplemented with 10 % FCS at 37 °C in 10 % CO_2_. HIV-1_GFP_ vectors were made and purified as described previously [50]. The A105S and the N74D mutation were introduced into pCMV∆R8.2 using the QuikChange II XL Kit (Stratagene) as previously described [24, 38]. Reverse transcriptase (RT) activity was measured by the Lenti-RT™ Activity Assay (Cavidi Tech, Uppsala, Sweden) following the manufacturer’s instructions. For infections, Jurkat cells were plated at 5 × 10^5^/mL in 48-well plates, infected with serial dilutions of HIV_GFP_ and analysed by FACS at the indicated time points. For DNA analysis, Jurkat cells were plated at 5 × 10^5^/mL in 6-well plates, infected at an MOI of 0.1 and DNA extracted 24, 48 h and 10 days later.

### qPCR

TaqMan qPCR was performed in an Eppendorf Realplex 2000 thermocycler as described [85]. For amplification of GFP (-strand) primers sequence was forward 5′-CAACAGCCACAACGTCTATATCAT-3′, reverse 5′-ATGTTGTGGCGGATCTTGAAG-3′, probe 5′-FAM-CCGACAAGCAGAAGAACGGCATCAA-TAMRA-3′. For amplification of 2LTR circular DNA, the same conditions were used with primers 2LTRqPCRF: 5′-AACTAGAGATCCCTCAGACCCTTTT-3′ and 2LTRqPCRRC: 5′-CTTGTCTTCGTTGGGAGTGAATT-3′ and probe 5′-FAM-CTAGAGTTTTCCACACTGAC-0-TAMRA-3′ [50]. Standards were prepared by PCR amplification of DNA from acutely infected cells with primers 2LTRF 5′-GCCTCAATAAAGCTTGCCTGG-3′ and 2LTRRC 5′-TCCCAGGCTCAGATCTGGTCTAAC-3′. The amplification product was cloned into TOPO vector, amplified and confirmed by sequencing. Alu-LTR Taqman qPCR was carried out as previously described [38] using primers ALU-forward, AAC TAG GGA ACC CAC TGC TTA AG and LTR1-reverse, TGC TGG GAT TAC AGG CGT GAG (for first round amplification) and ALU-forward AAC TAG GGA ACC CAC TGC TTA AG, LTR2-reverse, TGC TAG AGA TTT TCC ACA CTG ACT, ALU-probe, FAMRA—TAG TGT GTG CCC GTC TGT TGT GTG AC—TAM (for second round Taqman qPCR).

### Depletion of Nup153

Nup153 was depleted using shRNA expressed from a lentiviral vector. ShRNA oligonucleotides sequences were as follows: Nup153 forward 5′-GATCCGCAATTCGTCTCAAGCATTATTCAAGAGATAATGCTTGAGACGAATTGTTTTTTACGCGTG-3′, reverse 5′-AATTCACGCGTAAAAAACAATTCGTCTCAAGCATTATCTCTTGAATAATGCTTGAGACGAATTGCG-3′. Control shRNA targeting DsRed [50] mRNA was generated using the sequence: forward 5′-GATCCAAAAAGTAATGCAGAAGAAGACCATGGGCGAACCCATGGTCTTCTTCTGCATTAACGCGTG-3′, reverse 5′-AATTCACGCGTTAATGCAGAAGAAGACCATGGGTTCGCCCATGGTCTTCTTCTGCATTACTTTTTG-3′. Target and control DsRed oligonucleotides were from Sigma-Aldrich. Annealing was performed by incubation at 94 °C 30 s, 72 °C 2 min, 37 °C 2 min, 25 °C 2 min. Annealed oligonucleotides were inserted to Bam HI/EcoR I digested pSIREN-HIV (Clontech). The resulting construct was digested by Pst I/Xho I to release the puromycin resistant gene. The vector was then ligated with Sbf I/Xho I flanking the hygromycin resistant gene using Zero blunt PCR cloning kit (Life Technologies). After amplification and purification, vectors were confirmed by sequencing. Viral vectors were produced by Fugene transfection in 293T cells using plasmids pCMV∆R8.2 and pMD.G as described above and used to infect Jurkat hT5Cyp S322 or H126Q cells. Cells were selected in 250 μg/mL hygromycin for 1 week and clones were isolated by limiting dilution in 96 well plates. Nup153 depletion was confirmed by Western blot.

### CsA washout assays

Jurkat cells expressing T5Cyp S322 and H126Q have been previously described [60]. Cells were seeded in 96 well plates (100 μL final volume at 10^6^ cells/mL), drugs were added at a final concentration of 1 μM (CsA) or 3 μM (C-A1) and incubated on ice for 15 min in the dark. Plates were then incubated on ice for 15 min, virus was added and cells were spinoculated for 1.5 h, 1200×*g* at 4 °C. After spinoculation, media was replaced at different time points and cells were analyzed by flow cytometry 48 h post-infection. To calculate ToU_50_, the background infection detected in control samples (no CsA added) was subtracted and the percentage of GFP+ cells was plotted at each time point. Curve fitting was performed with software XLfit (ID Business Solutions Ltd.) using the Eadie-Hofstee algorithm for all data shown in Fig. 4.

### Cell fractionation

Cell fractionation was performed as previously described [50] with minor modifications. 10^7^ cells were washed once in 500 µL ice cold PBS and once in ice cold isotonic buffer (20 mM HEPES pH7.4, 110 mM KCL, 5 mM MgCl2, 0.5 mM EDTA, 1 mM DTT, 20 µg/mL aprotinin, 20 µg/mL leupeptin). Cells were gently re-suspended in 100 µL isotonic buffer and 1 mL isotonic buffer+ 0.5 % Igepal was added. Cells were incubated on ice for 5 min and gently mixed every minute by flicking before centrifugation for 2 min at 2000 rpm in a tabletop centrifuge at 4 °C. The supernatant was cleared by centrifugation at 13,000 rpm for 15 min at 4 °C (the cytoplasmic fraction). The pellet (the nuclear fraction) was washed once in isotonic buffer and re-suspended in 1 mL isotonic buffer. For Western blot of TRIMCyp, nuclear and cytoplasmic fractions were precipitated by adding 10 % (v/v) Trichloroacetic acid (TCA) followed by incubation at −20 °C for 16 h. Samples were centrifuged at 13,000 rpm for 30 min at 4 °C, washed twice with 500 µL ice-cold Acetone, dried and re-suspended in 60 μL isotonic buffer.

### Western blot

The following primary antibodies were used: anti-HIV-1 p24/p55 monoclonal antibodies EH12E1 and 3D3, mixed and diluted 1/300 (obtained from the AIDS repository reagent programme EVA centre for AIDS reagents, UK); rabbit polyclonal anti-Nup153 (Sigma-Aldrich HPA027896) diluted 1/2000, polyclonal anti-DNA Topoisomerase II (Topogen TG2011-1) diluted 1/500, mouse monoclonal anti-Na/K ATPase (Santa Cruz sc-58626) diluted 1/200, mouse monoclonal anti-Flag M2 (Sigma-Aldrich F3165) diluted 1/500. Samples were resuspended in 2× SDS loading buffer (0.5 M Tris HCl [pH 6.8], 1 % SDS, 10 % glycerol, 0.1 % bromophenol blue, 1 mM EDTA, 10 mM DTT, 20 µg of aprotinin/mL 2 µg of leupeptin hemisulfate/mL, 10 µg of phenylmethylsulfonyl fluoride) and the pH was adjusted to ~7.0 by addition of 1 µL of 1.5 M Tris–HCl (pH 8.8). Samples were resolved onto a 4–12 % gradient SDS-PAGE (pre-cast gel, Invitrogen) and transferred to a nitrocellulose membrane (Bio-Rad) [24]. Anti-mouse IgG HRP-conjugated antibodies were purchased from Dako (P0447) and diluted 1/5000 in 10 % non-fat milk (Tesco, London, UK); IgG HRP-conjugated anti-rabbit antibodies were also from Dako (P0448) and diluted 1/3000. Following transfer, the membrane was incubated with primary antibodies overnight at 4 °C and with secondary antibodies for 1 h at room temperature. Enhanced chemiluminescence (ECL Prime—Amersham) was used to develop the blots. Autoradiography films were exposed for different periods of time to ensure linearity of the signal.

### CPSF6 binding assays

Human 293T cells were transfected with plasmids expressing the CPSF6-FLAG protein. Twenty-four hours after transfection, cell were harvested, washed and resuspended in an hypotonic lysis buffer (10 mM Tris [pH 7.4], 1.5 mM MgCl_2_, 10 mM KCl, 0.5 mM dithiothreitol [DTT]). The cell suspension was incubated on ice for 10 min. Afterwards lysates were centrifuged at maximum speed in a refrigerated Eppendorf microcentrifuge (14,000×*g*) for 5 min. 4–5 μL of in vitro assembled HIV-1 capsid–nucleocapsid (CA–NC) complexes were incubated with 200 μl of cell lysates containing 0,1, 5 and 10 μM C-A1. Mixtures were incubated at room temperature for 1 h. 6 μM of PF74 was used as control. A fraction of these samples was stored (input). Mixtures were spun through a sucrose cushion (70 % sucrose, 1× PBS, 0.5 mM DTT) at 100,000×*g* in an SW55 rotor (Beckman) for 1 h at 4 °C. After centrifugation, the supernatant was carefully removed, and the pellets were resuspended in 1× SDS-PAGE loading buffer (pellet). The effect of C-A1 on CPSF6 binding to HIV-1 CA–NC complexes was determined by Western blotting using anti-FLAG antibodies. Levels of HIV-1 CA–NC complexes in the pellet was determined by Western blotting using anti-p24 CA antibodies. Fluorescent Wester b blotting allowed the quantification of bands in the 680 nm channels.

## Additional files

10.1186/s12977-016-0262-0 Docking of C-A1 into HIV-1 into hexameric capsid bound to PF-74 (4XFZpdb). Molecular docking was performed using the dock module within MOE software and the two subunits of capsid are depicted in pink and grey respectively. Key interactions between capsid and C-A1 (yellow) or PF74 (green) are depicted along with predicted hydrogen bonds and vdWaals contacts.
10.1186/s12977-016-0262-0 Diagrammatic representation of the CsA washout assay.
10.1186/s12977-016-0262-0 Conditions of the CsA washout assay. (A) T5Cyp and H126Q Jurkat cells were infected with increasing amounts of a WT HIV-1_GFP_ vector and analysed by FACS 36-48 h later to determine the level of restriction and the linearity of infection. The same titrations were performed using N74D (B) and A105S (C) viruses. (D) Table summarizing the titration results for panels (A, B and C); the percentage of GFP+ cells is shown for each virus dilution. (E) T5Cyp or H126Q cells were infected with WT HIV-1_GFP_ in the presence of the indicated doses of CsA and analysed by FACS 48 h later. (E) Samples in (E) were gated such that cells that did not fall within the forward/side scatter gate established for untreated (no CsA) cells were considered dead.
10.1186/s12977-016-0262-0 Conditions for the CsA washout assay. (A) H126Q cells were infected with WT HIV-1_GFP_ vector by spinoculation in the presence of CsA (1 μM) or C-A1 (3 μM). The drug was washed out at the indicated time points (time of washout) and cells were analysed by FACS 48 h later to determine the percentage of infected (GFP+) cells. (B) Same as (A) but T5Cyp cells expressing functional human TRIMCyp were used. (C) A prolonged time of uncoating assay, in which CsA was washed out at the indicated time points. Rescue of infection reaches a plateau after 10 h. Average ± SD of three independent experiments are shown in (A-C).
10.1186/s12977-016-0262-0 CsA washout assays in cells depleted of Nup153 or control DsRed cells. T5Cyp cells expressing an shRNA against Nup153 or DsRed (control) were infected at an MOI of 0.01–0.05 with an HIV-1_GFP_ vector (WT) in the presence of CsA (1 μM). The drug was washed out at the indicated time points (time of washout) and cells were analysed by FACS 48 h later to determine the percentage of infected (GFP+) cells. To control for specificity, cells were infected in the same way using the N74D mutant virus. Raw data of three independent experiments are shown. The data have been used to compile fold rescue levels shown in Figure 6.

## References

[1] Arhel N (2010). Revisiting HIV-1 uncoating. Retrovirology.

[2] Fassati A (2006). HIV infection of non-dividing cells: a divisive problem. Retrovirology.

[3] Forshey BM, von Schwedler U, Sundquist WI, Aiken C (2002). Formation of a human immunodeficiency virus type 1 core of optimal stability is crucial for viral replication. J Virol.

[4] Arfi V, Lienard J, Nguyen XN, Berger G, Rigal D, Darlix JL, Cimarelli A (2009). Characterization of the behavior of functional viral genomes during the early steps of human immunodeficiency virus type 1 infection. J Virol.

[5] Hulme AE, Perez O, Hope TJ (2011). Complementary assays reveal a relationship between HIV-1 uncoating and reverse transcription. Proc Natl Acad Sci USA.

[6] Yamashita M, Perez O, Hope TJ, Emerman M (2007). Evidence for direct involvement of the capsid protein in HIV infection of nondividing cells. PLoS Pathog.

[7] Lukic Z, Dharan A, Fricke T, Diaz-Griffero F, Campbell EM (2014). HIV-1 uncoating is facilitated by dynein and kinesin-1. J Virol.

[8] Xu H, Franks T, Gibson G, Huber K, Rahm N, De Castillia CS, Luban J, Aiken C, Watkins S, Sluis-Cremer N, Ambrose Z (2013). Evidence for biphasic uncoating during HIV-1 infection from a novel imaging assay. Retrovirology.

[9] Roa A, Hayashi F, Yang Y, Lienlaf M, Zhou J, Shi J, Watanabe S, Kigawa T, Yokoyama S, Aiken C, Diaz-Griffero F (2012). RING domain mutations uncouple TRIM5alpha restriction of HIV-1 from inhibition of reverse transcription and acceleration of uncoating. J Virol.

[10] Pawlica P, Berthoux L (2014). Cytoplasmic dynein promotes HIV-1 uncoating. Viruses.

[11] Yamashita M, Emerman M (2009). Cellular restriction targeting viral capsids perturbs human immunodeficiency virus type 1 infection of nondividing cells. J Virol.

[12] Qi M, Yang R, Aiken C (2008). Cyclophilin A-dependent restriction of human immunodeficiency virus type 1 capsid mutants for infection of nondividing cells. J Virol.

[13] Dismuke DJ, Aiken C (2006). Evidence for a functional link between uncoating of the human immunodeficiency virus type 1 core and nuclear import of the viral preintegration complex. J Virol.

[14] Tipper C, Sodroski J (2013). Enhanced autointegration in hyperstable simian immunodeficiency virus capsid mutants blocked after reverse transcription. J Virol.

[15] Schaller T, Ocwieja KE, Rasaiyaah J, Price AJ, Brady TL, Roth SL, Hue S, Fletcher AJ, Lee K, KewalRamani VN, Noursadeghi M, Jenner RG, James LC, Bushman FD, Towers GJ (2011). HIV-1 capsid–cyclophilin interactions determine nuclear import pathway, integration targeting and replication efficiency. PLoS Pathog.

[16] Lewinski MK, Yamashita M, Emerman M, Ciuffi A, Marshall H, Crawford G, Collins F, Shinn P, Leipzig J, Hannenhalli S, Berry CC, Ecker JR, Bushman FD (2006). Retroviral DNA integration: viral and cellular determinants of target-site selection. PLoS Pathog.

[17] Price AJ, Fletcher AJ, Schaller T, Elliott T, Lee K, Kewalramani VN, Chin JW, Towers GJ, James LC (2012). CPSF6 defines a conserved capsid interface that modulates HIV-1 replication. PLoS Pathog.

[18] Price AJ, Jacques DA, McEwan WA, Fletcher AJ, Essig S, Chin JW, Halambage UD, Aiken C, James LC (2014). Host cofactors and pharmacologic ligands share an essential interface in HIV-1 capsid that is lost upon disassembly. PLoS Pathog.

[19] Matreyek KA, Yucel SS, Li X, Engelman A (2013). Nucleoporin NUP153 phenylalanine-glycine motifs engage a common binding pocket within the HIV-1 capsid protein to mediate lentiviral infectivity. PLoS Pathog.

[20] Lee K, Ambrose Z, Martin TD, Oztop I, Mulky A, Julias JG, Vandegraaff N, Baumann JG, Wang R, Yuen W, Takemura T, Shelton K, Taniuchi I, Li Y, Sodroski J, Littman DR, Coffin JM, Hughes SH, Unutmaz D, Engelman A, KewalRamani VN (2010). Flexible use of nuclear import pathways by HIV-1. Cell Host Microbe.

[21] De Iaco A, Santoni F, Vannier A, Guipponi M, Antonarakis S, Luban J (2013). TNPO3 protects HIV-1 replication from CPSF6-mediated capsid stabilization in the host cell cytoplasm. Retrovirology.

[22] Di Nunzio F, Fricke T, Miccio A, Valle-Casuso JC, Perez P, Souque P, Rizzi E, Severgnini M, Mavilio F, Charneau P, Diaz-Griffero F (2013). Nup153 and Nup98 bind the HIV-1 core and contribute to the early steps of HIV-1 replication. Virology.

[23] Fricke T, Valle-Casuso JC, White TE, Brandariz-Nunez A, Bosche WJ, Reszka N, Gorelick R, Diaz-Griffero F (2013). The ability of TNPO3-depleted cells to inhibit HIV-1 infection requires CPSF6. Retrovirology.

[24] Zhou L, Sokolskaja E, Jolly C, James W, Cowley SA, Fassati A (2011). Transportin 3 promotes a nuclear maturation step required for efficient HIV-1 integration. PLoS Pathog.

[25] Peng K, Muranyi W, Glass B, Laketa V, Yant SR, Tsai L, Cihlar T, Muller B, Krausslich HG (2014). Quantitative microscopy of functional HIV post-entry complexes reveals association of replication with the viral capsid. Life.

[26] Hulme AE, Kelley Z, Foley D, Hope TJ (2015). Complementary assays reveal a low level of CA associated with viral complexes in the nuclei of HIV-1-infected cells. J Virol.

[27] Chin CR, Perreira JM, Savidis G, Portmann JM, Aker AM, Feeley EM, Smith MC, Brass AL (2015). Direct visualization of HIV-1 replication intermediates shows that capsid and CPSF6 modulate HIV-1 intra-nuclear invasion and integration. Cell Rep.

[28] Bhattacharya A, Alam SL, Fricke T, Zadrozny K, Sedzicki J, Taylor AB, Demeler B, Pornillos O, Ganser-Pornillos BK, Diaz-Griffero F, Ivanov DN, Yeager M (2014). Structural basis of HIV-1 capsid recognition by PF74 and CPSF6. Proc Natl Acad Sci USA.

[29] Bichel K, Price AJ, Schaller T, Towers GJ, Freund SM, James LC (2013). HIV-1 capsid undergoes coupled binding and isomerization by the nuclear pore protein NUP358. Retrovirology.

[30] Di Nunzio F, Danckaert A, Fricke T, Perez P, Fernandez J, Perret E, Roux P, Shorte S, Charneau P, Diaz-Griffero F, Arhel NJ (2012). Human nucleoporins promote HIV-1 docking at the nuclear pore, nuclear import and integration. PLoS ONE.

[31] Meehan AM, Saenz DT, Guevera R, Morrison JH, Peretz M, Fadel HJ, Hamada M, van Deursen J, Poeschla EM (2014). A cyclophilin homology domain-independent role for Nup358 in HIV-1 infection. PLoS Pathog.

[32] Christ F, Thys W, De Rijck J, Gijsbers R, Albanese A, Arosio D, Emiliani S, Rain JC, Benarous R, Cereseto A, Debyser Z (2008). Transportin-SR2 imports HIV into the nucleus. Curr Biol.

[33] Krishnan L, Matreyek KA, Oztop I, Lee K, Tipper CH, Li X, Dar MJ, Kewalramani VN, Engelman A (2010). The requirement for cellular transportin 3 (TNPO3 or TRN-SR2) during infection maps to human immunodeficiency virus type 1 capsid and not integrase. J Virol.

[34] Brass AL, Dykxhoorn DM, Benita Y, Yan N, Engelman A, Xavier RJ, Lieberman J, Elledge SJ (2008). Identification of host proteins required for HIV infection through a functional genomic screen. Science.

[35] De Iaco A, Luban J (2011). Inhibition of HIV-1 infection by TNPO3 depletion is determined by capsid and detectable after viral cDNA enters the nucleus. Retrovirology.

[36] Pizzato M, McCauley SM, Neagu MR, Pertel T, Firrito C, Ziglio S, Dauphin A, Zufferey M, Berthoux L, Luban J (2015). Lv4 is a capsid-specific antiviral activity in human blood cells that restricts viruses of the SIVMAC/SIVSM/HIV-2 lineage prior to integration. PLoS Pathog.

[37] Sowd GA, Serrao E, Wang H, Wang W, Fadel HJ, Poeschla EM, Engelman AN (2016). A critical role for alternative polyadenylation factor CPSF6 in targeting HIV-1 integration to transcriptionally active chromatin. Proc Natl Acad Sci USA.

[38] Vozzolo L, Loh B, Gane PJ, Tribak M, Zhou L, Anderson I, Nyakatura E, Jenner RG, Selwood D, Fassati A (2010). Gyrase B inhibitor impairs HIV-1 replication by targeting Hsp90 and the capsid protein. J Biol Chem.

[39] Blair WS, Pickford C, Irving SL, Brown DG, Anderson M, Bazin R, Cao J, Ciaramella G, Isaacson J, Jackson L, Hunt R, Kjerrstrom A, Nieman JA, Patick AK, Perros M, Scott AD, Whitby K, Wu H, Butler SL (2010). HIV capsid is a tractable target for small molecule therapeutic intervention. PLoS Pathog.

[40] Lamorte L, Titolo S, Lemke CT, Goudreau N, Mercier JF, Wardrop E, Shah VB, von Schwedler UK, Langelier C, Banik SS, Aiken C, Sundquist WI, Mason SW (2013). Discovery of novel small-molecule HIV-1 replication inhibitors that stabilize capsid complexes. Antimicrob Agents Chemother.

[41] Kelly BN, Kyere S, Kinde I, Tang C, Howard BR, Robinson H, Sundquist WI, Summers MF, Hill CP (2007). Structure of the antiviral assembly inhibitor CAP-1 complex with the HIV-1 CA protein. J Mol Biol.

[42] Tang C, Loeliger E, Kinde I, Kyere S, Mayo K, Barklis E, Sun Y, Huang M, Summers MF (2003). Antiviral inhibition of the HIV-1 capsid protein. J Mol Biol.

[43] Curreli F, Zhang H, Zhang X, Pyatkin I, Victor Z, Altieri A, Debnath AK (2011). Virtual screening based identification of novel small-molecule inhibitors targeted to the HIV-1 capsid. Bioorg Med Chem.

[44] Ternois F, Sticht J, Duquerroy S, Krausslich HG, Rey FA (2005). The HIV-1 capsid protein C-terminal domain in complex with a virus assembly inhibitor. Nat Struct Mol Biol.

[45] Thenin-Houssier S, de Vera IM, Pedro-Rosa L, Brady A, Richard A, Konnick B, Opp S, Buffone C, Fuhrmann J, Kota S, Billack B, Pietka-Ottlik M, Tellinghuisen T, Choe H, Spicer T, Scampavia L, Diaz-Griffero F, Kojetin DJ, Valente ST (2016). Ebselen, a small molecule capsid-inhibitor of HIV-1 replication. Antimicrob Agents Chemother.

[46] Shi J, Zhou J, Shah VB, Aiken C, Whitby K (2011). Small-molecule inhibition of human immunodeficiency virus type 1 infection by virus capsid destabilization. J Virol.

[47] Fricke T, Buffone C, Opp S, Valle-Casuso J, Diaz-Griffero F (2014). BI-2 destabilizes HIV-1 cores during infection and prevents binding of CPSF6 to the HIV-1 Capsid. Retrovirology.

[48] Kaplan SA (1970). Pharmacokinetic profile of coumermycin A. J Pharm Sci.

[49] Butler SL, Johnson EP, Bushman FD (2002). Human Immunodeficiency virus cDNA metabolism: notable stability of two-long terminal repeat circles. J Virol.

[50] Zaitseva L, Cherepanov P, Leyens L, Wilson SJ, Rasaiyaah J, Fassati A (2009). HIV-1 exploits importin 7 to maximize nuclear import of its DNA genome. Retrovirology.

[51] Pannecouque C, Pluymers W, Van Maele B, Tetz V, Cherepanov P, De Clercq E, Witvrouw M, Debyser Z (2002). New class of HIV integrase inhibitors that block viral replication in cell culture. Current biology : CB.

[52] Hazuda D, Iwamoto M, Wenning L (2009). Emerging pharmacology: inhibitors of human immunodeficiency virus integration. Annu Rev Pharmacol Toxicol.

[53] Gres AT, Kirby KA, KewalRamani VN, Tanner JJ, Pornillos O, Sarafianos SG (2015). STRUCTURAL VIROLOGY. X-ray crystal structures of native HIV-1 capsid protein reveal conformational variability. Science.

[54] Vilar S, Cozza G, Moro S (2008). Medicinal chemistry and the molecular operating environment (MOE): application of QSAR and molecular docking to drug discovery. Curr Top Med Chem.

[55] Pornillos O, Ganser-Pornillos BK, Yeager M (2011). Atomic-level modelling of the HIV capsid. Nature.

[56] Perez-Caballero D, Hatziioannou T, Zhang F, Cowan S, Bieniasz PD (2005). Restriction of human immunodeficiency virus type 1 by TRIM-CypA occurs with rapid kinetics and independently of cytoplasmic bodies, ubiquitin, and proteasome activity. J Virol.

[57] Hulme AE, Kelley Z, Okocha Z, Hope TJ (2005). Identification of capsid mutations that alter the rate of HIV-1 uncoating in infected cells. J Virol.

[58] Sayah DM, Sokolskaja E, Berthoux L, Luban J. Cyclophilin A retrotransposition into TRIM5 explains owl monkey resistance to HIV-1. Nature. 2004;430:569–73. 10.1038/nature02777 15243629

[59] Shi J, Friedman DB, Aiken C (2013). Retrovirus restriction by TRIM5 proteins requires recognition of only a small fraction of viral capsid subunits. J Virol.

[60] Neagu MR, Ziegler P, Pertel T, Strambio-De-Castillia C, Grutter C, Martinetti G, Mazzucchelli L, Grutter M, Manz MG, Luban J (2009). Potent inhibition of HIV-1 by TRIM5-cyclophilin fusion proteins engineered from human components. J Clin Investig.

[61] Sukegawa J, Blobel G (1993). A nuclear pore complex protein that contains zinc finger motifs, binds DNA, and faces the nucleoplasm. Cell.

[62] Cordes VC, Reidenbach S, Kohler A, Stuurman N, van Driel R, Franke WW (1993). Intranuclear filaments containing a nuclear pore complex protein. J Cell Biol.

[63] Walther TC, Fornerod M, Pickersgill H, Goldberg M, Allen TD, Mattaj IW (2001). The nucleoporin Nup153 is required for nuclear pore basket formation, nuclear pore complex anchoring and import of a subset of nuclear proteins. The EMBO J.

[64] Pante N, Bastos R, McMorrow I, Burke B, Aebi U (1994). Interactions and three-dimensional localization of a group of nuclear pore complex proteins. J Cell Biol.

[65] Shah S, Tugendreich S, Forbes D (1998). Major binding sites for the nuclear import receptor are the internal nucleoporin Nup153 and the adjacent nuclear filament protein Tpr. J Cell Biol.

[66] Makise M, Mackay DR, Elgort S, Shankaran SS, Adam SA, Ullman KS (2012). The Nup153-Nup50 protein interface and its role in nuclear import. J Biol Chem.

[67] Hase ME, Cordes VC (2003). Direct interaction with nup153 mediates binding of Tpr to the periphery of the nuclear pore complex. Mol Biol Cell.

[68] Paulillo SM, Phillips EM, Koser J, Sauder U, Ullman KS, Powers MA, Fahrenkrog B (2005). Nucleoporin domain topology is linked to the transport status of the nuclear pore complex. J Mol Biol.

[69] Diaz-Griffero F, Gallo DE, Hope TJ, Sodroski J (2011). Trafficking of some old world primate TRIM5alpha proteins through the nucleus. Retrovirology.

[70] Rasaiyaah J, Tan CP, Fletcher AJ, Price AJ, Blondeau C, Hilditch L, Jacques DA, Selwood DL, James LC, Noursadeghi M, Towers GJ (2013). HIV-1 evades innate immune recognition through specific cofactor recruitment. Nature.

[71] Naganuma T, Nakagawa S, Tanigawa A, Sasaki YF, Goshima N, Hirose T (2012). Alternative 3′-end processing of long noncoding RNA initiates construction of nuclear paraspeckles. EMBO J.

[72] Lambert SM, Langley DR, Garnett JA, Angell R, Hedgethorne K, Meanwell NA, Matthews SJ (2014). The crystal structure of NS5A domain 1 from genotype 1a reveals new clues to the mechanism of action for dimeric HCV inhibitors. Protein Sci Publ Protein Soc.

[73] Fassati A (2012). Multiple roles of the capsid protein in the early steps of HIV-1 infection. Virus Res.

[74] Schaller T, Ylinen LM, Webb BL, Singh S, Towers GJ (2007). Fusion of cyclophilin A to Fv1 enables cyclosporine-sensitive restriction of human and feline immunodeficiency viruses. J Virol.

[75] Yap MW, Dodding MP, Stoye JP (2006). Trim-cyclophilin A fusion proteins can restrict human immunodeficiency virus type 1 infection at two distinct phases in the viral life cycle. J Virol.

[76] Matreyek KA, Engelman A (2011). The requirement for nucleoporin NUP153 during human immunodeficiency virus type 1 infection is determined by the viral capsid. J Virol.

[77] Fassati A, Goff SP (2001). Characterization of intracellular reverse transcription complexes of human immunodeficiency virus type 1. J Virol.

[78] Ambrose Z, Aiken C (2014). HIV-1 uncoating: connection to nuclear entry and regulation by host proteins. Virology.

[79] Bestembayeva A, Kramer A, Labokha AA, Osmanovic D, Liashkovich I, Orlova EV, Ford IJ, Charras G, Fassati A, Hoogenboom BW (2015). Nanoscale stiffness topography reveals structure and mechanics of the transport barrier in intact nuclear pore complexes. Nat Nanotechnol.

[80] Marini B, Kertesz-Farkas A, Ali H, Lucic B, Lisek K, Manganaro L, Pongor S, Luzzati R, Recchia A, Mavilio F, Giacca M, Lusic M (2015). Nuclear architecture dictates HIV-1 integration site selection. Nature.

[81] Lelek M, Casartelli N, Pellin D, Rizzi E, Souque P, Severgnini M, Di Serio C, Fricke T, Diaz-Griffero F, Zimmer C, Charneau P, Di Nunzio F (2015). Chromatin organization at the nuclear pore favours HIV replication. Nat Commun.

[82] Rihn SJ, Wilson SJ, Loman NJ, Alim M, Bakker SE, Bhella D, Gifford RJ, Rixon FJ, Bieniasz PD (2013). Extreme genetic fragility of the HIV-1 capsid. PLoS Pathog.

[83] Chemical Computing Group, Molecular Operating Environment (MOE) 2014.09 2014, Chemical Computing Group Inc. 1010 Sherbooke St. West, Suite 910.

[84] Pornillos O, Ganser-Pornillos BK, Kelly BN, Hua Y, Whitby FG, Stout CD, Sundquist WI, Hill CP, Yeager M (2009). X-ray structures of the hexameric building block of the HIV capsid. Cell.

[85] Loh B, Vozzolo L, Mok BJ, Lee CC, Fitzmaurice RJ, Caddick S, Fassati A (2010). Inhibition of HIV-1 replication by isoxazolidine and isoxazole sulfonamides. Chem Biol Drug Des.

