# Supplementary figures and images for: HIV-1 capsid is involved in post-nuclear entry steps
Source: Retrovirology. 2016 Apr 23;13:28. doi: 10.1186/s12977-016-0262-0 (PMC4842275; doi:10.1186/s12977-016-0262-0)

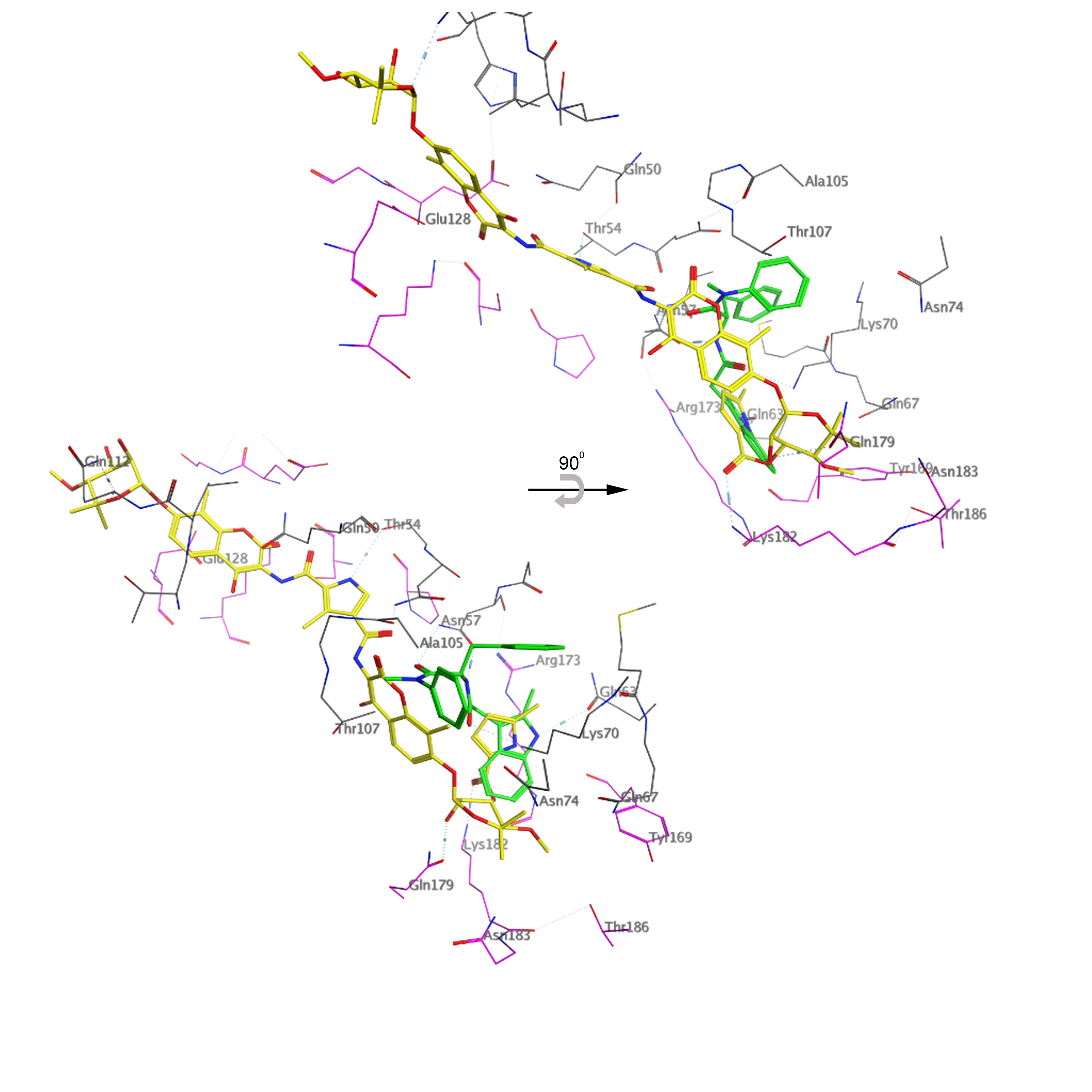

Supplement: Supplementary file 1 — 10.1186/s12977-016-0262-0 Docking of C-A1 into HIV-1 into hexameric capsid bound to PF-74 (4XFZpdb). Molecular docking was performed using the dock module within MOE software and the two subunits of capsid are depicted in pink and grey respectively. Key interactions between capsid and C-A1 (yellow) or PF74 (green) are depicted along with predicted hydrogen bonds and vdWaals contacts. [file 12977_2016_262_MOESM1_ESM.jpg]

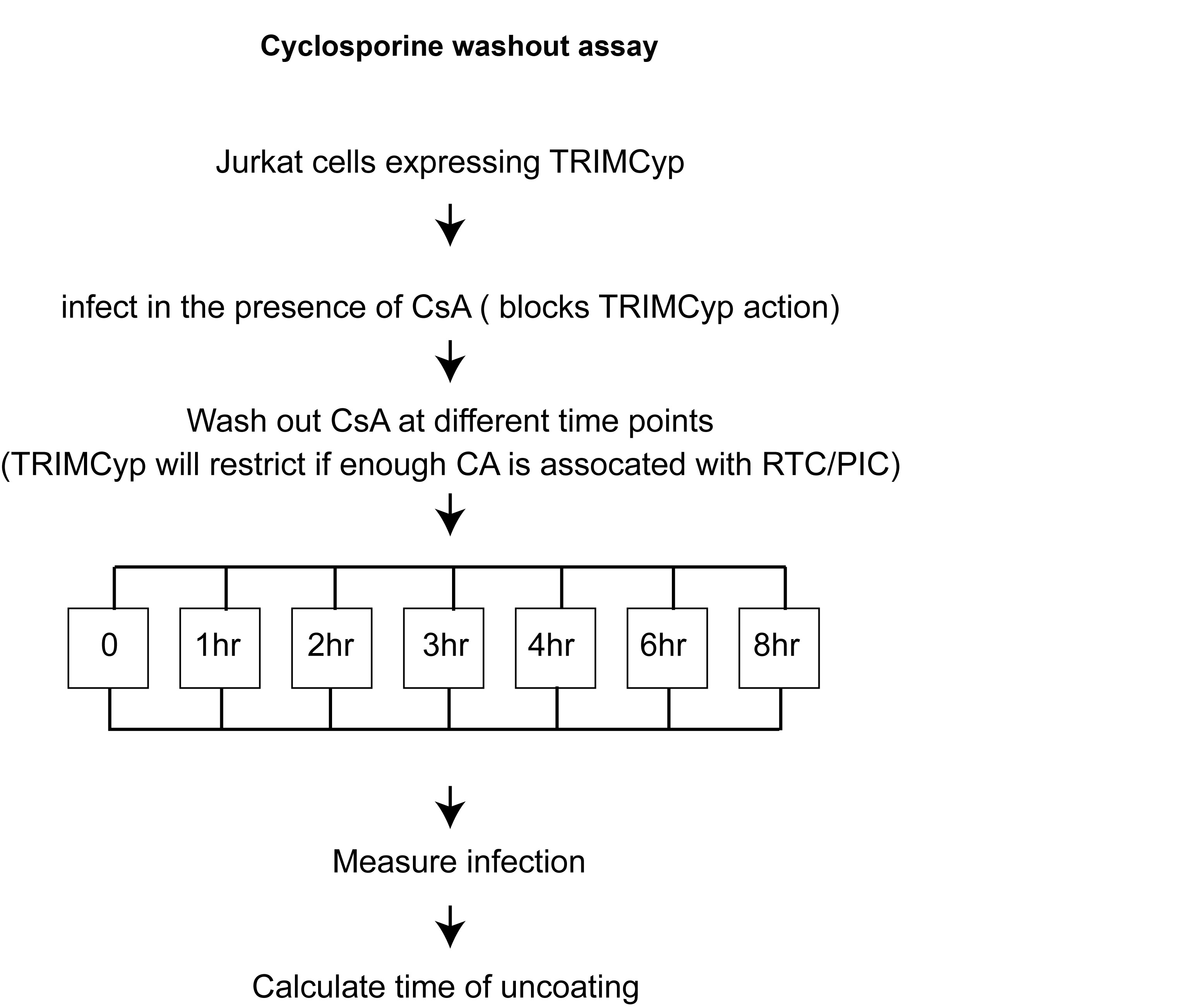

Supplement: Supplementary file 2 — 10.1186/s12977-016-0262-0 Diagrammatic representation of the CsA washout assay. [file 12977_2016_262_MOESM2_ESM.jpg]

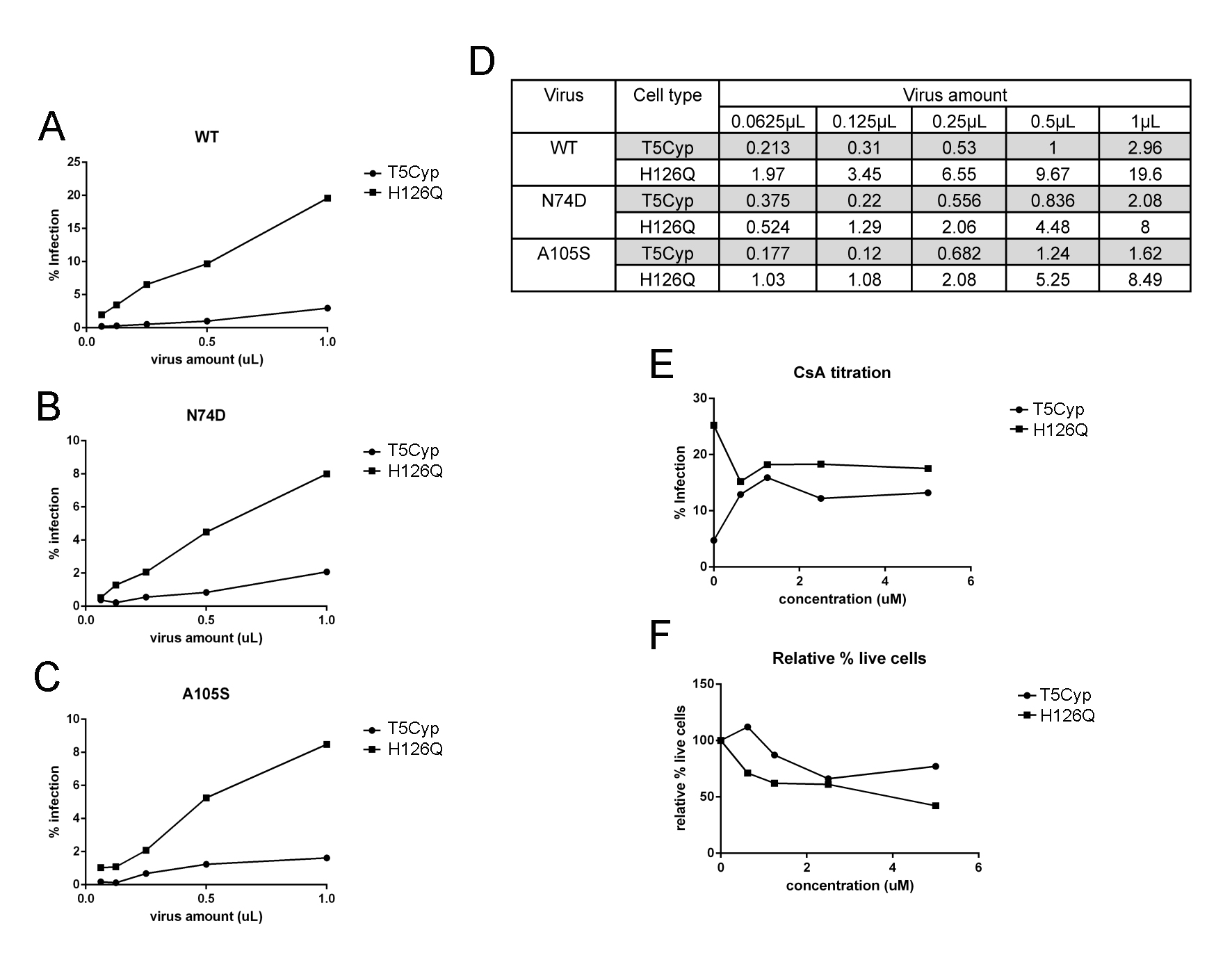

Supplement: Supplementary file 3 — 10.1186/s12977-016-0262-0 Conditions of the CsA washout assay. (A) T5Cyp and H126Q Jurkat cells were infected with increasing amounts of a WT HIV-1GFP vector and analysed by FACS 36-48 h later to determine the level of restriction and the linearity of infection. The same titrations were performed using N74D (B) and A105S (C) viruses. (D) Table summarizing the titration results for panels (A, B and C); the percentage of GFP+ cells is shown for each virus dilution. (E) T5Cyp or H126Q cells were infected with WT HIV-1GFP in the presence of the indicated doses of CsA and analysed by FACS 48 h later. (E) Samples in (E) were gated such that cells that did not fall within the forward/side scatter gate established for untreated (no CsA) cells were considered dead. [file 12977_2016_262_MOESM3_ESM.jpg]

**A**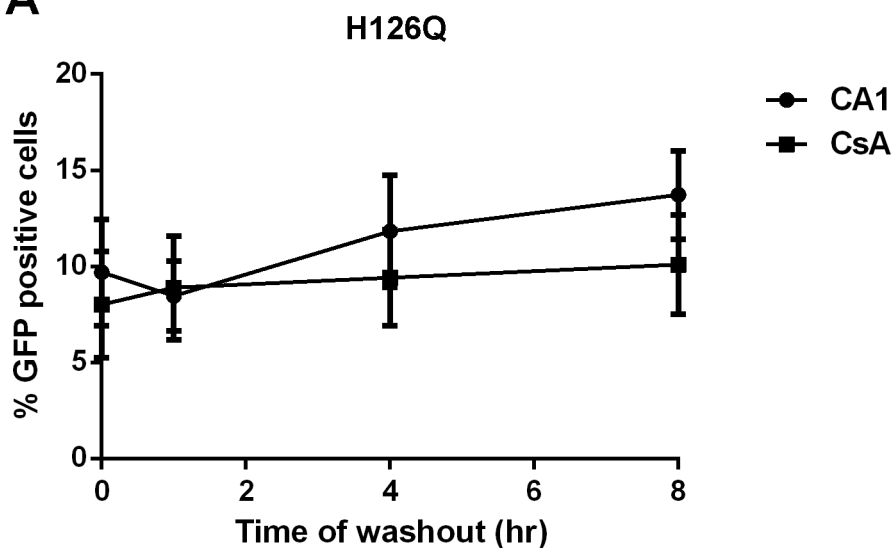**B**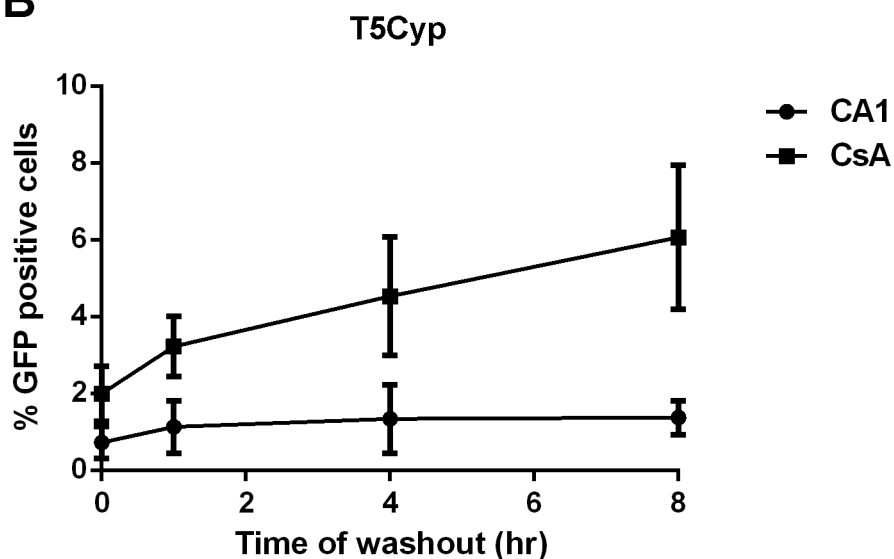**C**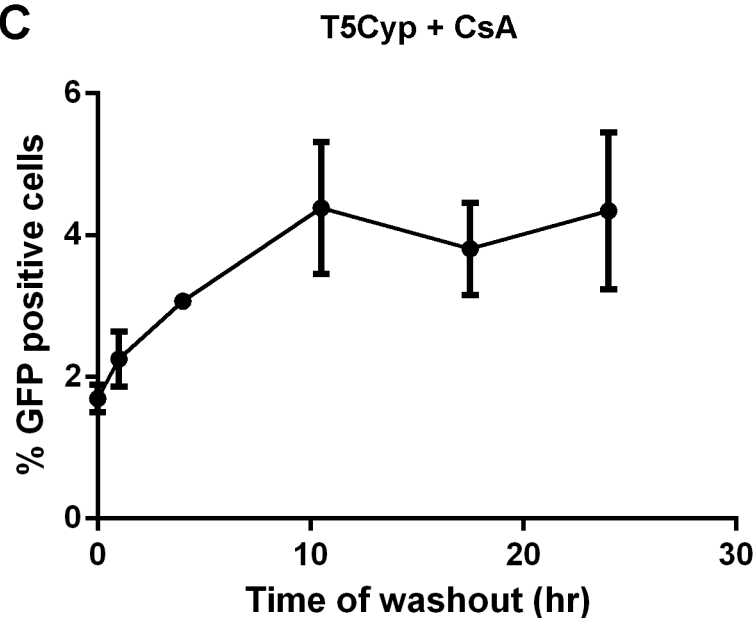

Supplement: Supplementary file 4 — 10.1186/s12977-016-0262-0 Conditions for the CsA washout assay. (A) H126Q cells were infected with WT HIV-1GFP vector by spinoculation in the presence of CsA (1 μM) or C-A1 (3 μM). The drug was washed out at the indicated time points (time of washout) and cells were analysed by FACS 48 h later to determine the percentage of infected (GFP+) cells. (B) Same as (A) but T5Cyp cells expressing functional human TRIMCyp were used. (C) A prolonged time of uncoating assay, in which CsA was washed out at the indicated time points. Rescue of infection reaches a plateau after 10 h. Average ± SD of three independent experiments are shown in (A-C). [file 12977_2016_262_MOESM4_ESM.pdf]

WT

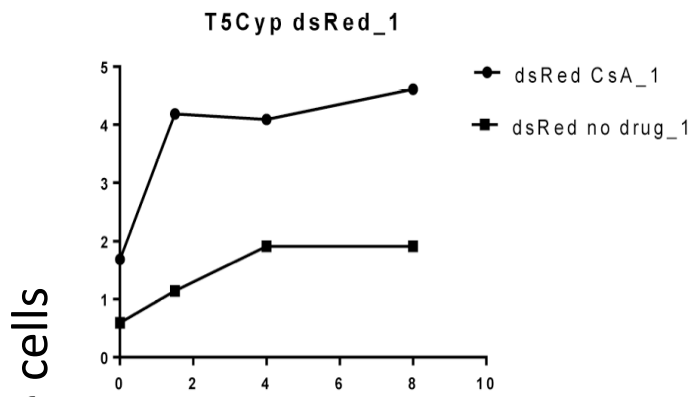

N74D

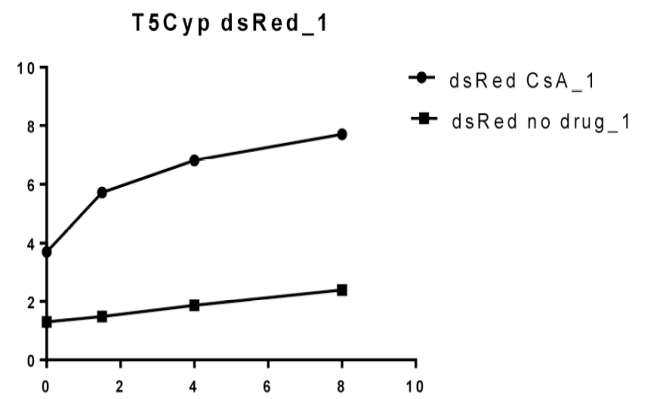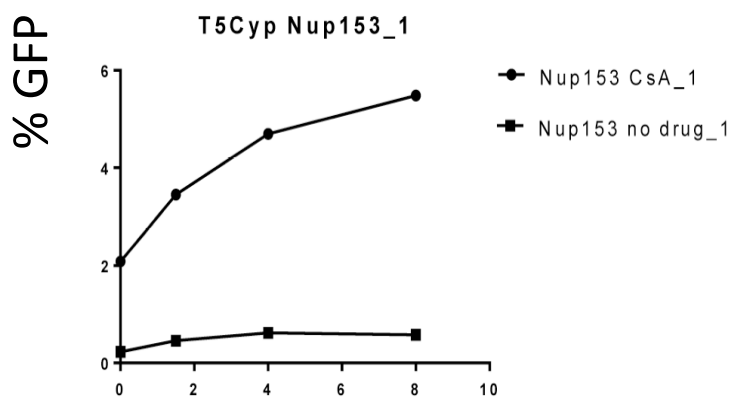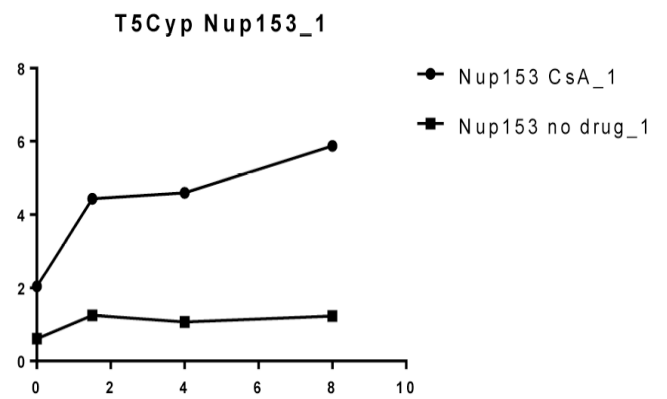

Time of Washout (hr)

WT

N74D

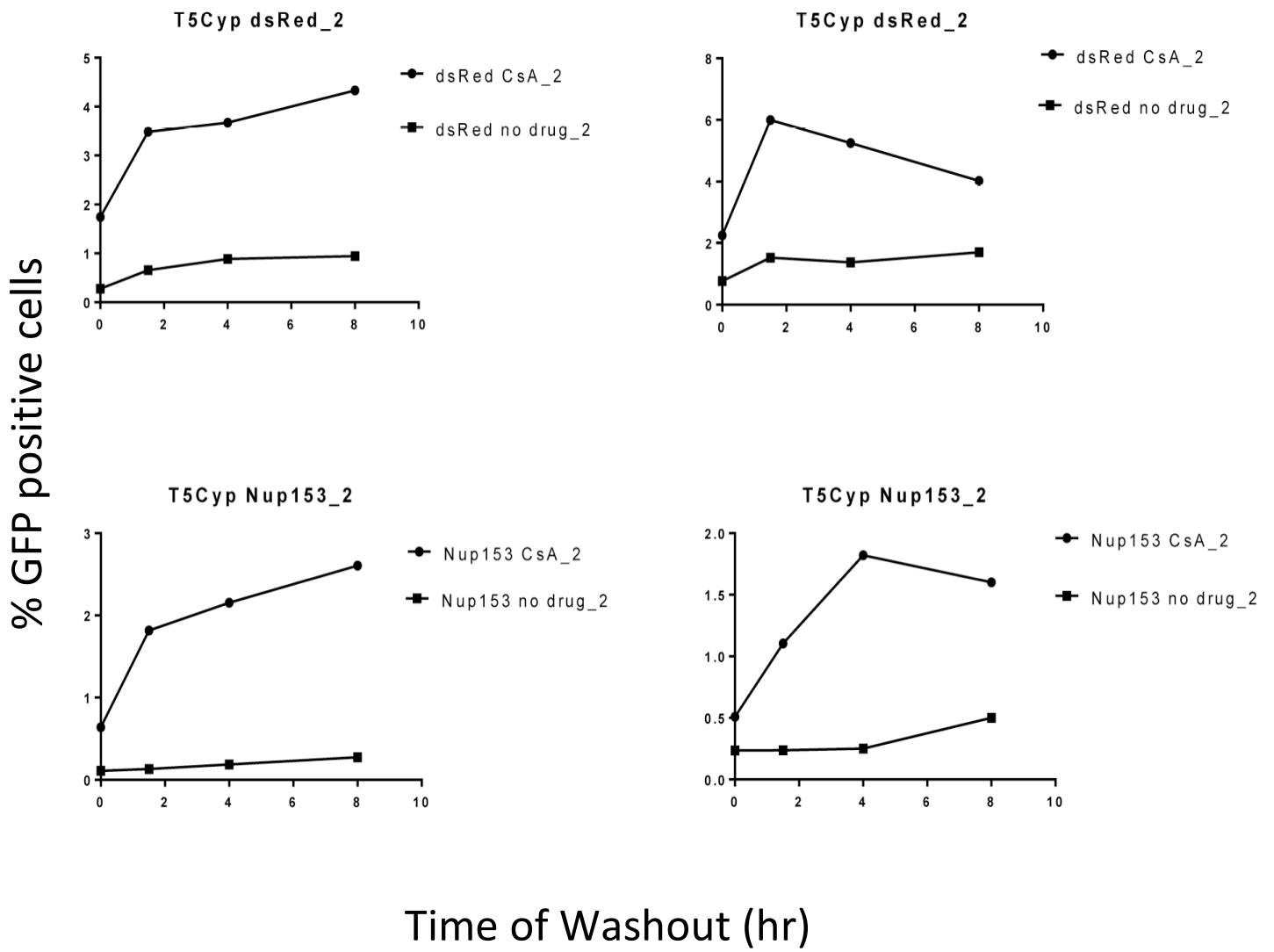

WT

N74D

% GFP positive cells

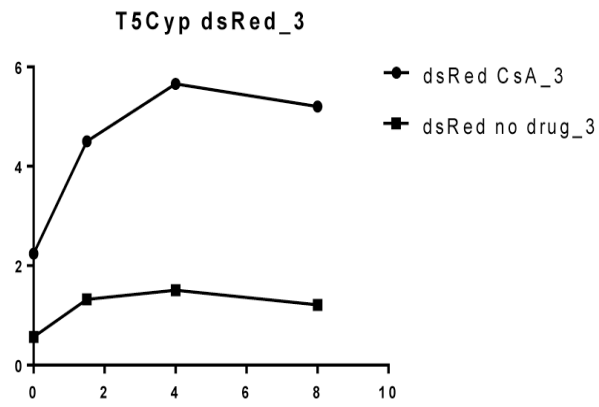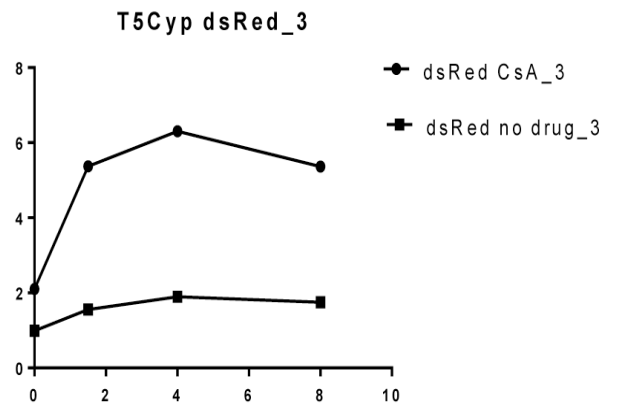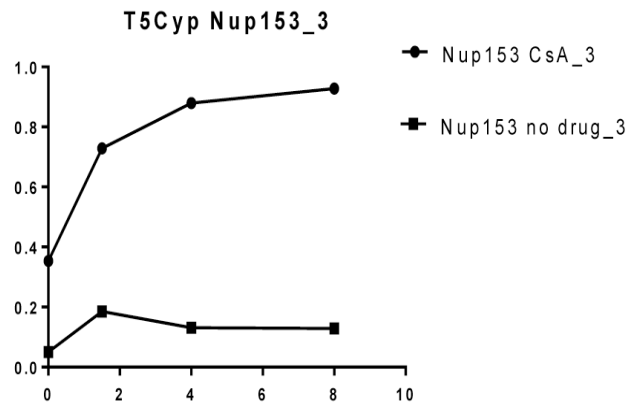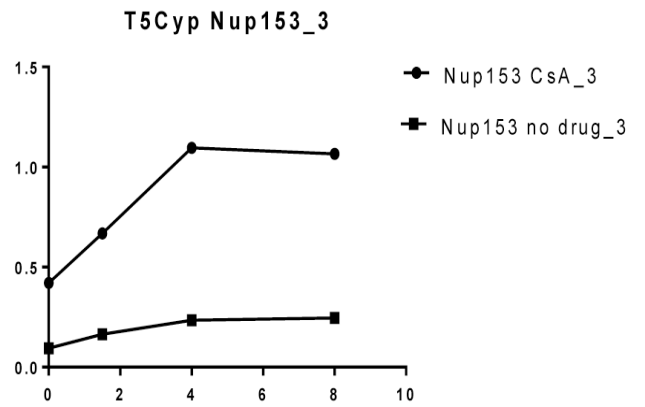

Time of Washout (hr)

Supplement: Supplementary file 5 — 10.1186/s12977-016-0262-0 CsA washout assays in cells depleted of Nup153 or control DsRed cells. T5Cyp cells expressing an shRNA against Nup153 or DsRed (control) were infected at an MOI of 0.01–0.05 with an HIV-1GFP vector (WT) in the presence of CsA (1 μM). The drug was washed out at the indicated time points (time of washout) and cells were analysed by FACS 48 h later to determine the percentage of infected (GFP+) cells. To control for specificity, cells were infected in the same way using the N74D mutant virus. Raw data of three independent experiments are shown. The data have been used to compile fold rescue levels shown in Figure 6. [file 12977_2016_262_MOESM5_ESM.pdf]
